# Enhanced Catalytic Performance for H_2_ Harvesting from Steam Reforming of Methanol Using Glycine Nitrate Process Synthesized Novel CuFeO_2_–ZnFe_2_O_4_ Porous Nanocomposite Catalyst

**DOI:** 10.1021/acs.langmuir.5c00895

**Published:** 2025-06-04

**Authors:** Chung-Lun Yu, Subramanian Sakthinathan, Ching-Lung Chen, Satoshi Kameoka, Naratip Vittayakorn, Hongbing Jia, Te-Wei Chiu

**Affiliations:** † Department of Materials and Mineral Resources Engineering, 34877 National Taipei University of Technology, Taipei 10608, Taiwan; ‡ Institute of Materials Science and Engineering, National Taipei University of Technology, Taipei 10608, Taiwan; § High-value Biomaterials Research and Commercialization Center, National Taipei University of Technology, Taipei 10608, Taiwan; ∥ Department of Safety, Health and Environmental Engineering, 56082 Ming Chi University of Technology, New Taipei City 243303, Taiwan; ⊥ Center for Environmental Sustainability and Human Health, Ming Chi University of Technology, New Taipei City 243303, Taiwan; # Center for Sustainability and Energy Technologies, Chang Gung University, Taoyuan 33302, Taiwan; ∇ Institute of Multidisciplinary Research for Advanced Materials, 92125 Tohoku University, Sendai 980-8577, Japan; ○ Advanced Materials Research Unit, School of Science, King Mongkut’s Institute of Technology Lad Krabang, Bangkok 10520, Thailand; ◆ Key Laboratory for Soft Chemistry and Functional Materials of Ministry of Education, Nanjing University of Science and Technology, Nanjing 210094, China

## Abstract

Nowadays, depleting petrochemical resources and global fossil fuel pollution are urgent issues. Hydrogen (H_2_) has emerged as a promising alternative energy source to combat climate change, the energy crisis, and environmental concerns. However, in the hydrogen energy sector, the storage and transportation of H_2_ remain challenging. The industrial H_2_ production path involves the use of steam reforming of methanol, which could effectively avoid the danger of directly using H_2_. Methanol steam reforming (SRM) offers a safe and practical route for H_2_ production, leveraging methanol-favorable properties. In this work, a CuFeO_2_–ZnFe_2_O_4_ nanocomposite with enhanced surface area was synthesized via the glycine–nitrate process (GNP) and employed as a catalyst for SRM. Structural and morphological analyses were conducted using X-ray diffraction studies, field emission scanning electron microscopy, transmission electron microscopy, Raman spectroscopy, and BET. The as-combusted nanocomposite exhibited a specific surface area increase from 1.90 to 6.32 m^2^/g. The best performance achieved was an H_2_ production rate of 6984 ± 35 mL STP min^–1^ g-cat^–1^ (or) 312 ± 2 mmol STP min^–1^g-cat^–1^ with a flow rate of 30 sccm at 500 °C, without activation treatment. Based on the establishment, highlight the potential of CuFeO_2_–ZnFe_2_O_4_ nanocomposite as a cost-effective catalyst for on-demand hydrogen generation in fuel cell applications in the future.

## Graphical abstract

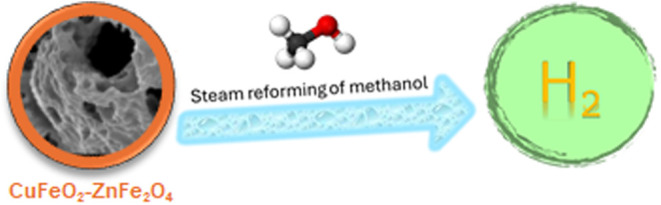

## 1 Introduction

Hydrogen is expected to be a highly economical and ideal environmentally friendly energy source due to its lack of toxicity and carbon emissions, carbonate production, and highly efficient energy conversion. Hydrogen energy is an important clean energy source because of its high energy density (143 MJ kg^–1^) and low carbon emissions. Hydrogen is a great energy carrier, but unfortunately, its production from naturally occurring substances is quite expensive. Currently, almost all hydrogen is produced from fossil fuels, accounting for approximately 6 and 2% of global natural gas and coal consumption, respectively.^1,2^ Moreover, 95% of H_2_ is produced from the reforming of natural gases, but this process also results in large emissions of CO_2_. In addition, the rapid development of industry and technology have led to an energy crisis and extreme disasters, including the rapid depletion of fossil fuels, climate change, and the greenhouse effect.^3,4^

Given the current trends in environmental change, the next generation of alternative energy is a serious global issue with great potential for future development.^5^ However, hydrogen energy presents challenges that need to be considered, such as the efficient production of hydrogen, storage media, and transportation safety. Furthermore, the maintenance of hydrogen refueling and storage facilities is crucial for hydrogen-based fuel cells.^6^ The issues remaining to be addressed include the high production costs, postcompletion maintenance, safety concerns, and difficulties in storing hydrogen. The safe storage of hydrogen is crucial due to its flammability and wide range of combustibility in air as compared with natural gas. Fuel cells are the most promising development for replacing traditional fuel in vehicles owing to their high hydrogen energy conversion. At present, direct hydrogen production entails high transportation costs and low volumetric energy density, as well as security risks during storage. Additionally, a hydrogen leak is more dangerous than a natural gas leak. Therefore, the development of safe hydrogen storage technology is of paramount importance.^7^

In recent years, there has been significant interest in various hydrogen generation processes, including thermochemical hydrogen,^8^ solar to hydrogen,^9^ water electrolysis,^10^ water gas shift reaction, and steam reforming.^11,12^ Among those aforementioned hydrogen production processes, steam reforming is particularly noteworthy due to the low reforming temperatures and low concentrations of CO in products, so it is used as a simple and efficient hydrogen production process and commonly applied in the industrial field. Of the various alternative fuels available, methanol stands out due to its high hydrogen–carbon ratio. Moreover, methanol has an advantage over other fuel sources for hydrogen production due to the single carbon atom contained in the structure.^13,14^ As the single carbon atom lacks a C=C bond, the use of methanol as a hydrogen source could effectively decrease the production of coke. Moreover, the chemical stability of methanol could eliminate safety concerns during transportation as compared with those surrounding hydrogen, which is highly flammable in air at concentrations of 4 to 75%.^15^ Among various hydrogen production methods, each approach presents unique advantages and limitations. Water electrolysis, for example, offers high-purity hydrogen with zero CO_2_ emissions when powered by renewable electricity; however, its energy consumption is relatively high (∼50–60 kWh/kg-H_2_), and the capital cost of electrolyzers remains a major barrier for large-scale adoption.^16,17^ Thermochemical water splitting using metal oxides is another CO_2_-free method, but it requires extremely high operating temperatures (>1000 °C), complex reactor design, and challenges in cyclic material stability.^6^ The water–gas shift reaction (WGS) is widely used in industrial H_2_ purification following reforming. Yet, it is not a standalone H_2_ source and relies on upstream syngas production, usually from fossil fuels.^9^ In contrast, steam reforming of methanol (SRM) combines relatively mild operating conditions (200–300 °C), high H_2_ selectivity, and liquid-fuel safety, making it an attractive method for on-demand hydrogen generation in portable or automotive applications.^12^ Moreover, methanol high H/C ratio and low sulfur content reduce the risk of catalyst poisoning and carbon deposition.

Steam reforming of methanol is a hydrogen production process that has been explored and studied by researchers who have investigated the reaction mechanism under the typical operating parameters (1 bar and H_2_O/methanol = 1–3). The aforementioned studies have broadly divided the mechanisms into 3 possible patterns: (1) parallel reaction, (2) intermediate product conversion, and (3) decomposition reaction.^18^

1. In parallel reaction, steam reforming of methanol (SRM) involves one main reaction and two side reactions. The main reaction is the steam reforming (SR) endothermic ([Disp-formula eq1]). Methanol (CH_3_OH) and steam react to yield H_2_ and carbon dioxide (CO_2_). Parallel reaction also involves two side reactions: methanol decomposition (MD) ([Disp-formula eq2]) and the water gas shift reaction (WGSr) ([Disp-formula eq3]).^19^ The reaction formulas are as follows:
1.1 $${\mathrm{CH}}_{3}\mathrm{OH}+H_{2}O⇄{\mathrm{CO}}_{2}+3H_{2}$$
1.2 $${\mathrm{CH}}_{3}\mathrm{OH}⇄2H_{2}+\mathrm{CO}$$
1.3 $$\mathrm{CO}+H_{2}O⇄{\mathrm{CO}}_{2}+H_{2}$$
2. In intermediate product conversion, the reaction generates a methoxyl group and produces an intermediate product, either formate (HCOOCH_3_) or formaldehyde (HCHO). Based on the methoxyl generation, adsorption, and intermediate conversion. This mechanism allows control over the overall process in terms of the conversion efficiency and reaction efficiency of the SRM reaction.^20,21^ Below are their reactions ([Disp-formula eq4]) to ([Disp-formula eq9]):
2.1 $$2{\mathrm{CH}}_{3}\mathrm{OH}⇄{\mathrm{HCOOCH}}_{3}+2H_{2}$$
2.2 $${\mathrm{HCOOCH}}_{3}+2H_{2}O⇄2\mathrm{HCOOH}+2H_{2}$$
2.3 $$\mathrm{HCOOH}⇄{\mathrm{CO}}_{2}+H_{2}$$
2.4 $${\mathrm{CH}}_{3}\mathrm{OH}⇄\mathrm{HCHO}+H_{2}$$
2.5 $$\mathrm{HCHO}+H_{2}O⇄\mathrm{HCOOH}+{2H}_{2}$$
2.6 $$\mathrm{HCOOH}⇄{\mathrm{CO}}_{2}+H_{2}$$
3. In thermal decomposition, the SRM reaction only includes two reactions: the ([Disp-formula eq10]) MD and ([Disp-formula eq11]) WGSr reactions.^22^ The reaction formulas are as follows:
3.1 $${\mathrm{CH}}_{3}\mathrm{OH}⇄{2H}_{2}+\mathrm{CO}$$
3.2 $$\mathrm{CO}+H_{2}O⇄{\mathrm{CO}}_{2}+H_{2}$$

The SRM process follows the MD reaction ([Disp-formula eq10]) and WGSr reaction ([Disp-formula eq11]), as described by researchers. However, the CO concentration of this method of production is lower than the theoretical prediction analyzed by mass spectrometry, making this mechanism controversial.^23^

Moreover, compared to the hydrogen production rate of the SR reaction, that of the MD reaction is lower. Meanwhile, side reactions occur easily when the ratio of the steam-to-methanol is lowered. Under this condition, the side reactions, resulting can result in the production of many byproducts, including methane, methyl formate (HCO_2_CH_3_), dimethyl ether ((CH_3_)_2_O), etc.^12^

Recently, the catalysts used in the SRM process have usually consisted of Cu, Zn, Cr, Fe, and Ni single metal-based or composite catalysts.^24–27^ The different elements and different fabrication processes can affect the performance of the catalysts. In previous studies, high stability and high-efficiency Zn-based catalysts and high activity and high hydrogen selectivity Cu-based catalysts have usually been selected for use as the catalysts in the SRM process.^28,29^ However, both Cu-based and Zn-based catalysts have disadvantages. The thermal stability and long-term use of Cu-based catalysts are challenging to apply to the SRM process. In addition, the reaction temperature range of Cu-based catalysts is narrow, so the process is prone to inactivation. On the other hand, Zn-based catalysts have an operation limit, as they are only active at high temperatures. Metal oxide promoters (such as ZnO, ZrO_2_, CeO_2_, Al_2_O_3_, etc.) have been applied to solve the challenges posed by the catalysts used in SRM and improve catalysis. The incorporation of a promoter could effectively increase the catalytic activity and thermal stability.^30–32^

Binary metal oxides (BMOs) have a wide range of applications across various fields, offering distinct advantages over single metal oxides. The combination of different metal elements in BMOs leads to synergistic effects, resulting in enhanced theoretical capacitance, improved electrical conductivity, superior magnetism, increased catalytic activity, and a greater number of active sites. Additionally, their composition, synthesis parameters, band gap, surface area, and crystallinity can be tailored to suit specific applications. Therefore, BMOs demonstrate greater thermal and chemical stability, making them particularly well-suited for reactor-based catalyst materials. Besides, controlling the morphology, size, and composition of BMOs during synthesis presents significant challenges that can affect their overall performance. The complex interactions between different metal elements further complicate the understanding and prediction of material properties. Advanced characterization techniques are often necessary to assess the structure, composition, and interfacial properties accurately. Furthermore, many synthesis methods developed at the lab scale are difficult to scale up cost-effectively, and interfacial defects or mismatched crystal structures can lead to diminished functionality.^33–35^

In addition, the use of Zn-based and Cu-based catalyst composites could effectively offset the individual disadvantages of the catalysts and enhance their value. Another way to improve the properties of the catalysts is to reduce them to nanosized powders. In such nanosized catalysts, the specific surface area (SSA) is increased, which also increases the number of active sites on the surface of the catalysts.^36,37^ The aforementioned literature includes several nanoparticulation processes for catalyst preparation, including a solution combustion method, a hydrothermal method, and electron spinning, among others.^38–40^ Of these, the glycine nitrate process (GNP) is simple, has rapid process steps, is low in cost, and yields a product with high catalytic activity due to its porous structure. Chiu et al. studied Cu-based delafossite catalysts (CuCrO_2_, CuFeO_2_, etc.) and Zn-based spinel catalysts (ZnCr_2_O_4_ and ZnFe_2_O_4_) fabricated by the GNP method and applied them to the SRM process.^32,41,42^ The combination of CuFeO_2_ and ZnFe_2_O_4_ demonstrates a synergistic effect that enhances key material characteristics such as lattice parameter (Å), the crystallite size (nm), and microstrain. Moreover, their similar crystal structures contribute to the formation of a stable composite with improved mechanical strength, catalytic efficiency, structural stability, and electron transfer properties. Additionally, interfacial interactions between the two phases can reduce recombination and allow them to function effectively as cocatalysts.^43,44^ Hence, the catalysts prepared by GNP manifested as nanosized particles, which were estimated to be 20–50 nm in size. Based on the above, a nanosized metal oxide porous powder prepared via the GNP method could be a potential catalyst for application in fuel cells and hydrogen production by steam reforming.

## 2 Experimental Procedure

### 2.1 The Preparation Procedure of CuFeO_2_–ZnFe_2_O_4_ Porous Nanocomposite

The CuFeO_2_–ZnFe_2_O_4_ nanocomposite was prepared by the GNP process and denoted as CFO-ZFO. Metal nitrates (Cu, Zn, and Fe) were used as the starting reagents. The molar ratio of the glycine to metal nitrate was set at 1.7. Furthermore, the molar ratios of copper nitrate, zinc nitrate, and iron nitrate were *X*:1–*X*:2–*X* (*X* = 0–1), respectively. All samples had ratios of CuFeO_2_ (named CFO) to ZnFe_2_O_4_ (named ZFO) in gradations of 10% (i.e., 100:0 (100%), 90:10 (90%), 80:20 (80%), 70:30 (70%), 60:40 (60%), 50:50 (50%), 40:60 (40%), 30:70 (30%), 20:80 (20%), 10:90 (10%), and 0:100 (0%)). As shown in Figure 1, the initial reagents for the CuFeO_2_–ZnFe_2_O_4_ nanocomposite precursor were dissolved in deionized water and stirred at 80 °C until a clear, precipitate-free solution was formed. The CuFeO_2_–ZnFe_2_O_4_ nanocomposites precursor was then dried in an oven at 80 °C for 12 h until it reached a gel-like consistency. Finally, the precursor-gel CuFeO_2_–ZnFe_2_O_4_ nanocomposites were ignited on a hot plate at 300 °C.

**Figure 1:**
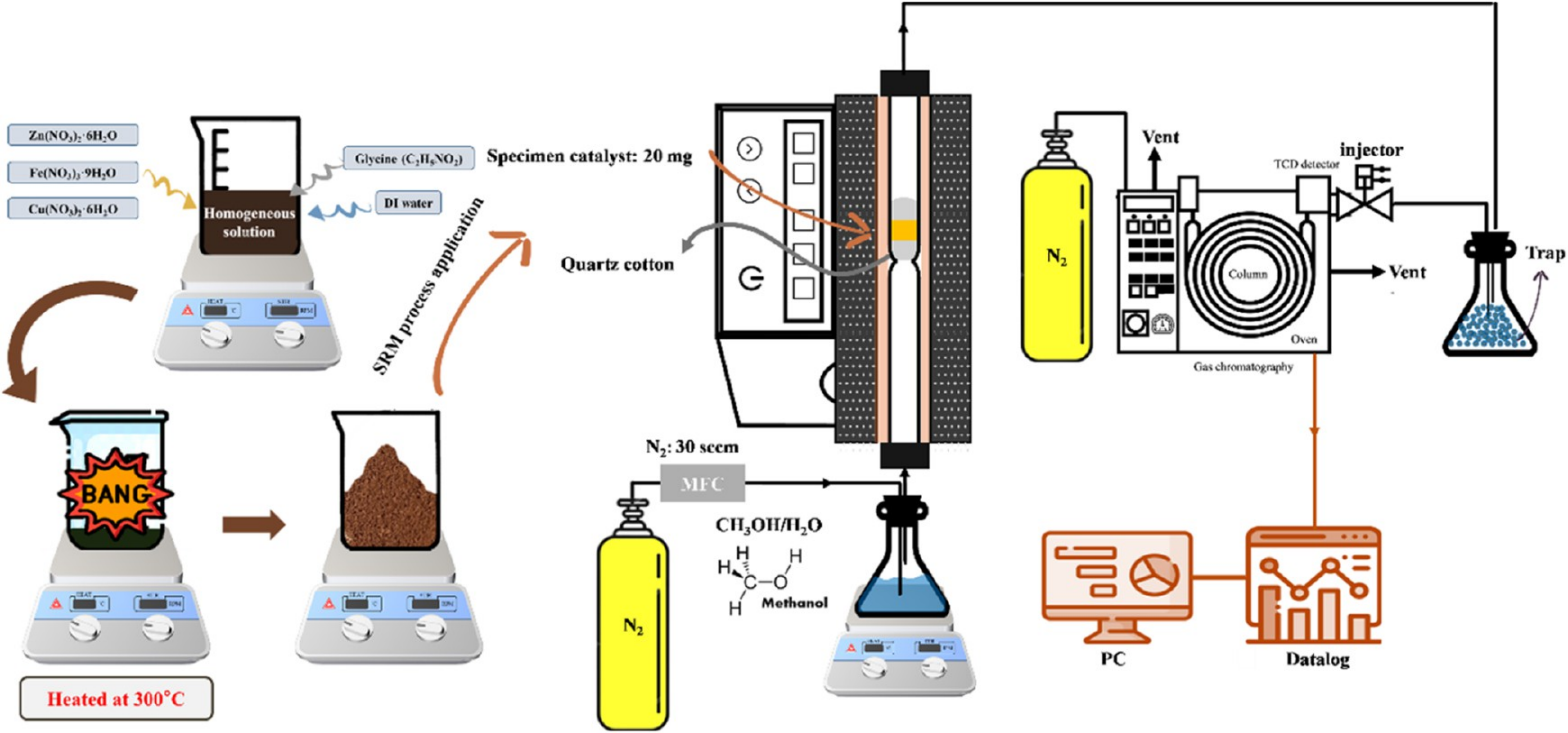
Flowchart of the CuFeO_2_–ZnFe_2_O_4_ nanocomposites preparation and H_2_ production by steam reforming of methanol.

During the early phase of the GNP method, the solution underwent boiling, dehydration, and foaming, ultimately culminating in self-sustained combustion that yielded a porous powder. The heat generated during this exothermic reaction was sufficient to drive the synthesis of the CuFeO_2_–ZnFe_2_O_4_ nanocomposites. Notably, this combustion reaction proceeded spontaneously after ignition without requiring continuous external heating. Owing to the high temperatures involved in the GNP method, the as-prepared product could be applied directly in catalytic applications without the need for further treatment.

### 2.2 Materials and Characterizations

The reagents used in this study, including copper nitrate hexahydrate [Cu­(NO_3_)_2_·6H_2_O], zinc nitrate hexahydrate [Zn­(NO_3_)_2_·6H_2_O], iron­(III) nitrate nonahydrate [Fe­(NO_3_)_3_·9H_2_O], and glycine [C_2_H_5_NO_2_], were obtained from SHOWA and Sigma-Aldrich. The characteristics of the CuFeO_2_–ZnFe_2_O_4_ nanocomposite were measured with the appropriate analytical instruments. A D2 Phaser X-ray diffractometer (XRD, Bruker) equipped with Cu Kα radiation (λ = 1.5418 Å) and operated at a working voltage of 30 kV and working current of 10 mA was used to study the crystallinity of the CuFeO_2_–ZnFe_2_O_4_ nanocomposite. The scanning range of the XRD measurement was fixed at 20°–80°. Furthermore, the phase composition of the nanocomposite was determined by MDI JADE 5.0 software with the JCPDS database. The microscopic morphology of the porous catalysts was measured with a Regulus 8100 field emission scanning electron microscope (FESEM, HITACHI). Selected area electron diffraction (SAED) patterns were investigated with a JEM2100F transmission electron microscope (TEM, JEOL) operated at 10^–8^ Pa and a 200 kV working voltage. The elemental distribution of the porous catalysts was determined with an energy-dispersive X-ray (EDX) spectroscope mounted on the TEM instrument. For the ACRON microscopic Raman spectroscopy (UniNanoTech Co., Ltd.), a 532 nm laser was used as the excitation source to observe the vibrational pattern of the specimen. Brunauer identified the specific surface area–Emmett–Teller method using a Tristar II 3030 analyzer (BET, Micromeritics). In this work, the hydrogen temperature-programmed reduction (H_2_-TPR) profile of CuFeO_2_–ZnFe_2_O_4_ nanocomposite was used to investigate the reduction condition and recorded with a TCA 2004A Thermoconductivity Analyzer (China Chromatography). X-ray photoelectron spectroscopy was recorded by a JPS-9030 X-ray photoelectron spectrometer (XPS, JEOL), and the charge calibration was compared against that of carbon (C 1s, 284.6 eV).

### 2.3 Catalyst and Stability Test

The catalyst test and stability evaluation of the CuFeO_2_–ZnFe_2_O_4_ nanocomposite were performed in a fixed-bed reactor (quartz tube) with an inner diameter of 1.2 cm. The system flowchart is depicted in Figure 1, which shows the gas cylinder, tube furnace, methanol steam, datalogger, and gas chromatograph. The carrier gas used in the process was N_2_, which was used to convey methanol–water vapor with a fixed molar ratio of CH_3_OH to H_2_O of 1:1, and the inlet partial pressures of methanol, water, and N_2_ read 30.2, 31.0, and 35.2 kPa, respectively (GHSV of methanol–water mixture vapor: 36,000 h^–1^). The CuFeO_2_–ZnFe_2_O_4_ nanocomposite was weighed into samples of 20 mg and placed in a tubular furnace equipped with thermocouples. A GC 1000 gas chromatograph (GC, China Chromatography) with a carbon molecular sieve column (60/80 Carboxen 1000) was used to measure CO, CO_2_, H_2_, and CH_4_ (7 ft 1/16 in, stainless steel), and a thermal conductivity detector was used during the SRM process. The standard of GC calibration gas was obtained via (Air Liquide Far Eastern). Catalytic activity was evaluated by measuring the H_2_ concentration, and gas chromatography was utilized to calculate the H_2_ productivity (mL STP min^–1^ g-cat^–1^). The catalytic performance of the catalyst was determined with the conversion rate of CH_3_OH, H_2_ production, and selectivity as indexes. The calculation method was as follows, and the “*n*” mentioned in the eq 12 to 14 is the mole of the gas product, and selectivity was calculated eq 15 to 16.^19,45–47^

4.1 $$m\mathrm{ethanol conversion}\;\left(\%\right)=\frac{\left(methanol\right)in-(methanol)out}{\left(methanol\right)in}\times 100$$

4.2 $$H_{2}\;\mathrm{production rate}=\frac{H_{2}\;concentration\times flow\;rate\;of\;methano\mathrm{l/water}\;mixture}{catalyst\;volume\times catalyst\;weight}$$

4.3 $$H_{2}\;\mathrm{selectivity}\;\left(\%\right)=\frac{nH_{2}}{{(nH}_{2}+nCH_{4})}\times 100$$

4.4 $$\mathrm{CO}\;\mathrm{selectivity}\;\left(\%\right)=\frac{n\mathrm{CO}}{{(n\mathrm{CO}+n\mathrm{CO}}_{2}+nCH_{4})}\times 100$$

4.5 $${\mathrm{CO}}_{2}\;\mathrm{selectivity}\;\left(\%\right)=\frac{n{CO}_{2}}{{(nCO+nCO}_{2}+nCH_{4})}\times 100$$

4.6 $${\mathrm{CH}}_{4}\;\mathrm{selectivity}\;\left(\%\right)=\frac{nCH_{4}}{{(nCO+nCO}_{2}+nCH_{4})}\times 100$$

## 3 Results and Discussion

To identify the crystal phase of the as-combusted nanocomposite, the crystallinity of the CuFeO_2_–ZnFe_2_O_4_ nanocomposites was studied by XRD measurement. The XRD patterns of the CuFeO_2_–ZnFe_2_O_4_ nanocomposites are presented in Figure 2. As shown in the diffraction patterns of the CuFeO_2_–ZnFe_2_O_4_ nanocomposites indicated that crystallized CuFeO_2_ and ZnFe_2_O_4_ exhibited the rhombohedral and cubic phases, respectively. The immiscibility of CuFeO_2_–the nonshifting peaks in the patterns judged ZnFe2O4 nanocomposite. The characteristic diffraction peaks of the CuFeO_2_ rhombohedral phase (PDF#39-0246) were at 31.2, 35.6, 40.2, 55.2, and 61.0°, corresponding to the (006), (012), (104), (018), and (110) planes; the characteristic diffraction peaks of the ZnFe_2_O_4_ cubic phase (PDF#22-1012) were at 30.2, 35.6, 43.3, 53.7, 57.2, and 62.9°, corresponding to the (220), (311), (400), (422), (511) and (440) planes. The curve showed the highly crystallized CuFeO_2_ rhombohedral phase (black line) and ZnFe_2_O_4_ cubic phase (dark blue line). In addition, small amounts of CuO and Cu_2_O were present, as indicated by the diffraction peak immediately after the (311) peak. Furthermore, no major impurity peaks were observed in the XRD patterns of the CuFeO_2_–ZnFe_2_O_4_ nanocomposites. The XRD patterns of the CuFeO_2_–ZnFe_2_O_4_ nanocomposites after the SRM process are shown in Figure 3. Metallic Cu^0^ and iron carbides (Fe_3_C and Fe_5_C_2_) were revealed by the XRD patterns of the CuFeO_2_–ZnFe_2_O_4_ nanocomposites after the SRM process. Meanwhile, during the steam reforming process, graphitic carbon was formed and covered the surface. This graphitic carbon attached to the surface of the CuFeO_2_–ZnFe_2_O_4_ nanocomposites, which reacted with the Fe ion, reducing the nanocomposites from CuFeO_2_ and ZnFe_2_O_4_ to form iron carbides. In contrast, the CuFeO_2_–ZnFe_2_O_4_ nanocomposites after SRM treatment exhibited characteristic diffraction peaks of metallic Cu^0^ (PDF#85-1326) at 43.1 and 50.4°, corresponding to the (111) and (200) crystal planes, respectively. Based on this, the Cu^1+^ ions on the CuFeO_2_ surface were reduced to Cu^0^ and attached to the catalysts.

**Figure 2:**
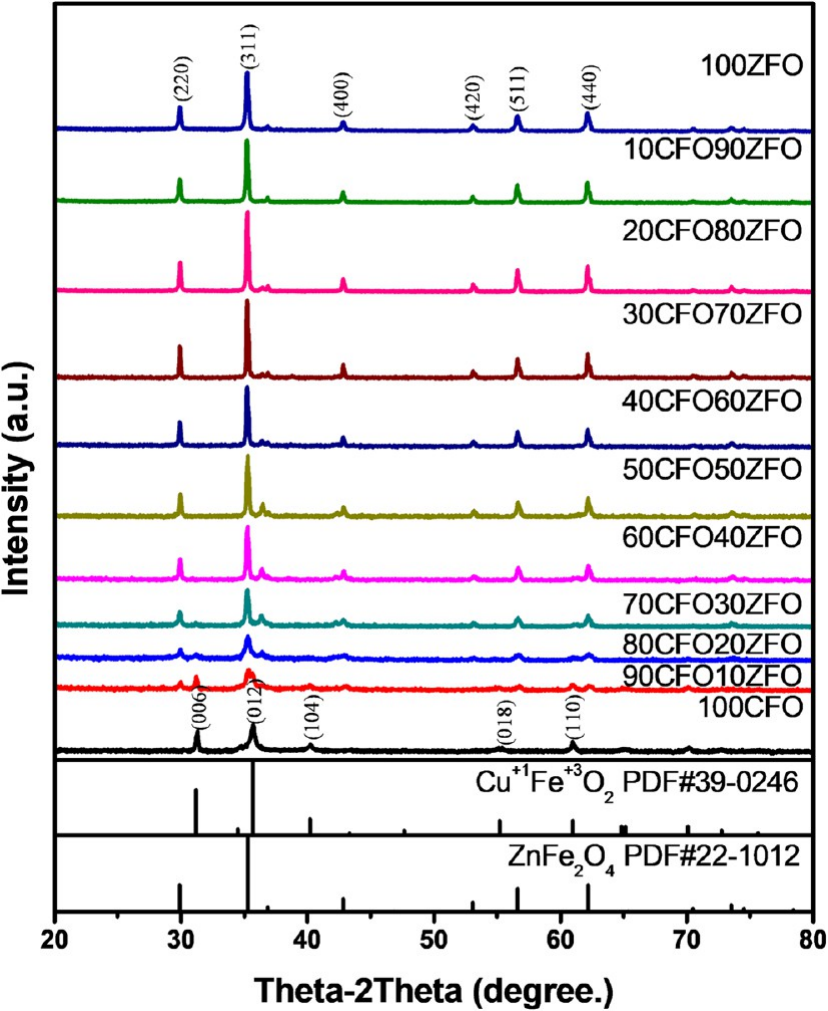
XRD patterns of CuFeO_2_, 90CuFeO_2_-10ZnFe_2_O_4_, 80CuFeO_2_-20ZnFe_2_O_4_, 70CuFeO_2_-30ZnFe_2_O_4_, 60CuFeO_2_–40ZnFe_2_O_4_, 50CuFeO_2_-50ZnFe_2_O_4_, 40CuFeO_2_-60ZnFe_2_O_4_, 30CuFeO_2_-70ZnFe_2_O_4_, 20CuFeO_2_-80ZnFe_2_O_4_, 10CuFeO_2_-90ZnFe_2_O_4_, and ZnFe_2_O_4_ nanocomposites prepared by the GNP method.

**Figure 3:**
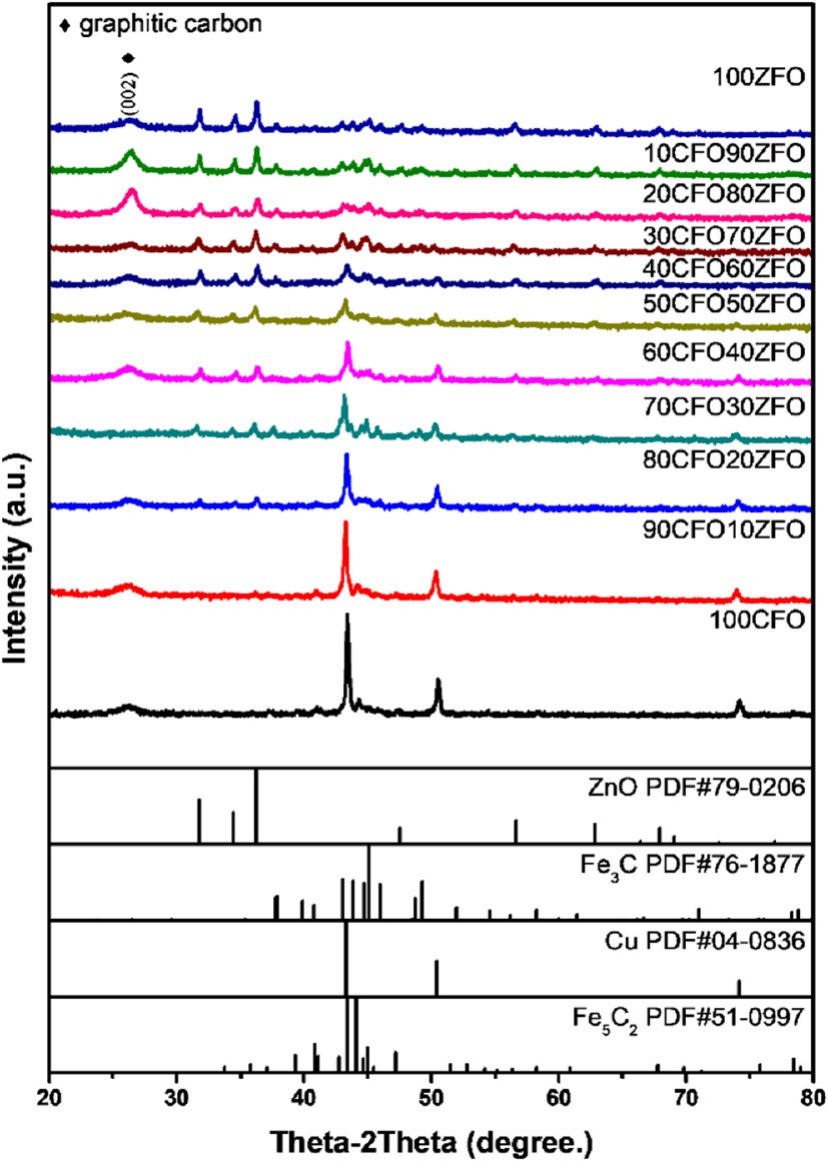
XRD patterns of Figure 2. XRD patterns of CuFeO_2_, 90CuFeO_2_-10ZnFe_2_O_4_, 80CuFeO_2_-20ZnFe_2_O_4_, 70CuFeO_2_-30ZnFe_2_O_4_, 60CuFeO_2_–40ZnFe_2_O_4_, 50CuFeO_2_-50ZnFe_2_O_4_, 40CuFeO_2_-60ZnFe_2_O_4_, 30CuFeO_2_-70ZnFe_2_O_4_, 20CuFeO_2_-80ZnFe_2_O_4_, 10CuFeO_2_-90ZnFe_2_O_4_, and ZnFe_2_O_4_ nanocomposites prepared by the GNP method after the SRM process.

FESEM recorded the microscopic morphology of the CuFeO_2_–ZnFe_2_O_4_ nanocomposites. Figure 4 shows the FESEM images of (a) CuFeO_2_, (b) 90CuFeO_2_-10ZnFe_2_O_4_, (c) 80CuFeO_2_-20ZnFe_2_O_4_, (d) 70CuFeO_2_-30ZnFe_2_O_4_, (e) 60CuFeO_2_–40ZnFe_2_O_4_, (f) 50CuFeO_2_-50ZnFe_2_O_4_, (g) 40CuFeO_2_-60ZnFe_2_O_4_, (h) 30CuFeO_2_-70ZnFe_2_O_4_, (i) 20CuFeO_2_-80ZnFe_2_O_4_, (j) 10CuFeO_2_-90ZnFe_2_O_4_, and (k) ZnFe_2_O_4_ porous powders. As can be seen in Figure 4, the FESEM images of the CuFeO_2_–ZnFe_2_O_4_ nanocomposites exhibited a coral-like porous structure with a high specific surface area because of the large amount of gas generated and released during the GNP reaction. Figure 5 shows FESEM images of (a) CuFeO_2_, (b) 90CuFeO_2_-10ZnFe_2_O_4_, (c) 80CuFeO_2_-20ZnFe_2_O_4_, (d) 70CuFeO_2_-30ZnFe_2_O_4_, (e) 60CuFeO_2_–40ZnFe_2_O_4_, (f) 50CuFeO_2_-50ZnFe_2_O_4_, (g) 40CuFeO_2_-60ZnFe_2_O_4_, (h) 30CuFeO_2_-70ZnFe_2_O_4_, (i) 20CuFeO_2_-80ZnFe_2_O_4_, (j) 10CuFeO_2_-90ZnFe_2_O_4_ and (k) ZnFe_2_O_4_ porous powders after the SRM process. In Figure 5, it can be seen that the CuFeO_2_–ZnFe_2_O_4_ nanocomposites had some filamentous and granular particles that precipitated and covered the surface. Based on the XRD results in Figure 3, the filamentous and granular particles that precipitated were determined to be iron carbides that formed from carbon deposition, and the iron reduced from CuFeO_2_ and ZnFe_2_O_4_ during the SRM process. Furthermore, the porous structure of the CuFeO_2_–ZnFe_2_O_4_ nanocomposites remained after the SRM process, revealing the stability of the external structure.

**Figure 4:**
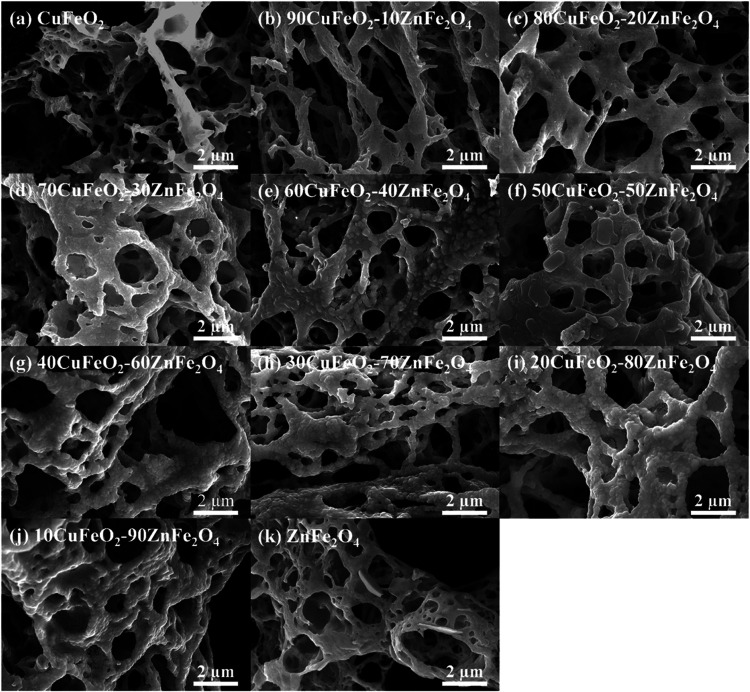
FESEM images of CuFeO_2_–ZnFe_2_O_4_ nanocomposites prepared by the GNP method.

**Figure 5:**
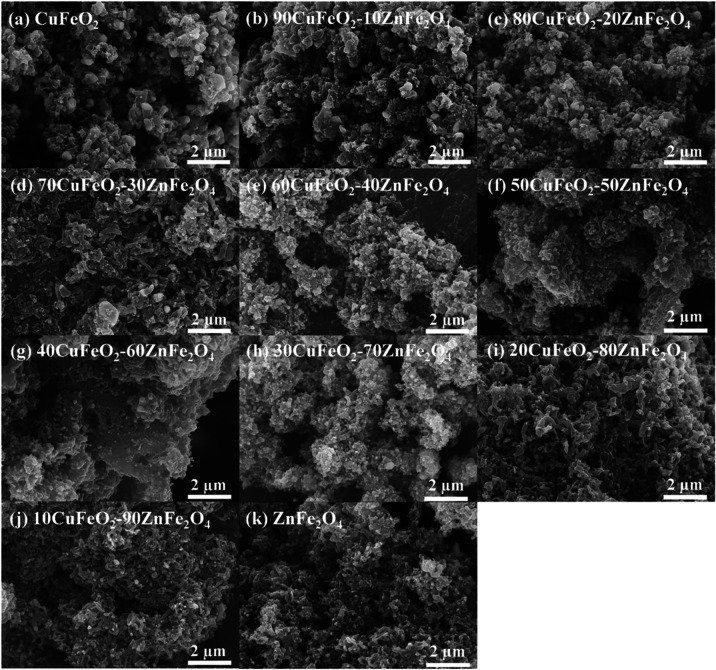
FESEM images of CuFeO_2_–ZnFe_2_O_4_ nanocomposites prepared by the GNP method after the SRM process.

Figure 6 presents TEM images of the 60CuFeO_2_–40ZnFe_2_O_4_ nanocomposite prepared by the GNP method. In Figure 6a,b, the 60CuFeO_2_–40ZnFe_2_O_4_ nanocomposite revealed a porous structure because of the amount of gas released during the preparation reaction. The insert in Figure 6c shows an HRTEM image of the 60CuFeO_2_–40ZnFe_2_O_4_ nanocomposite, from which the particle size was estimated to be around 20–30 nm. Figure 6d shows the SAED pattern of the 60CuFeO_2_–40ZnFe_2_O_4_ nanocomposite, which was consistent with the diffraction spectra of the same nanocomposite. Figure 6e,f shows the 60CuFeO_2_–40ZnFe_2_O_4_ nanocomposite after the SRM process. As shown in Figure 6e,f, the particles retained the porous structure. Therefore, the results of the TEM images were consistent with the FESEM images. The elemental distributions of the 60CuFeO_2_–40ZnFe_2_O_4_ nanocomposite before and after SRM were examined by STEM-EDX spectroscopy, and the results are shown in Figure 7. As can be seen in Figure 7a–f, the 60CuFeO_2_–40ZnFe_2_O_4_ nanocomposite revealed even distribution of the elements. Furthermore, in Figure 8a–g, after the SRM process, the surface of the 60CuFeO_2_–40ZnFe_2_O_4_ nanocomposite was covered with a layer of carbon, which corresponded to the XRD result presented in Figure 3. Figure 9a–d presents the TEM/SAED diffraction patterns of the 60CuFeO_2_–40ZnFe_2_O_4_ nanocomposite. Figure 9a presents the TEM/SAED diffraction patterns of the 60CuFeO_2_–40ZnFe_2_O_4_ nanocomposite (CuFeO_2_ part), and Figure 9b, the simulated diffraction pattern of CuFeO_2_. As evidenced in Figure 9a,b, the phase of CuFeO_2_ (PDF# 39-0246) was well crystallized, and the zone axis was [100]. Figure 9c the TEM/SAED diffraction patterns of the 60CuFeO_2_–40ZnFe_2_O_4_ nanocomposite (ZnFe_2_O_4_ part), and Figure 9d, the simulated diffraction pattern of ZnFe_2_O_4_. As can be seen in Figure 9c,d, the phase of ZnFe_2_O_4_ (PDF# 22-1012) was well crystallized, and the zone axis was [01̅1]. Based on the SAED diffraction patterns, the crystalline structure of the 60CuFeO_2_–40ZnFe_2_O_4_ nanocomposite was identified, and the results corresponded to those of the XRD studies.

**Figure 6:**
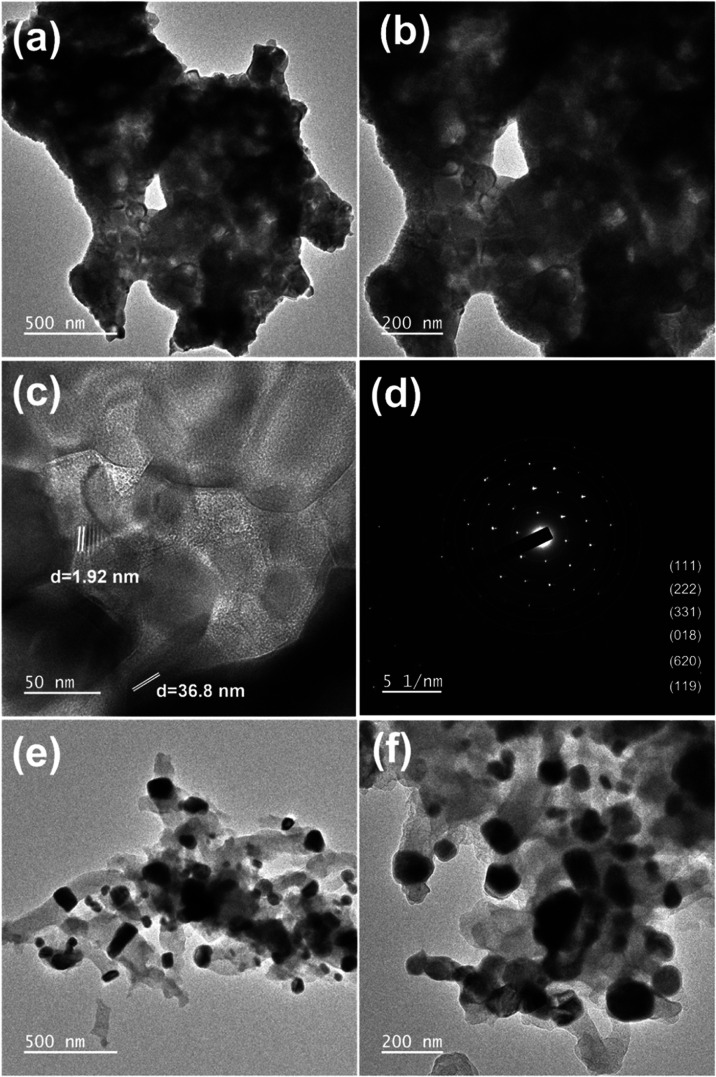
TEM images of (a, b) 60CuFeO_2_–40ZnFe_2_O_4_ nanocomposite prepared by the GNP method, (c) Lattice fringe of 60CuFeO_2_–40ZnFe_2_O_4_ nanocomposite, (d) SAED pattern of 60CuFeO_2_–40ZnFe_2_O_4_ nanocomposite, (e, f) HRTEM images of 60CuFeO_2_–40ZnFe_2_O_4_ nanocomposite after SRM process.

**Figure 7:**
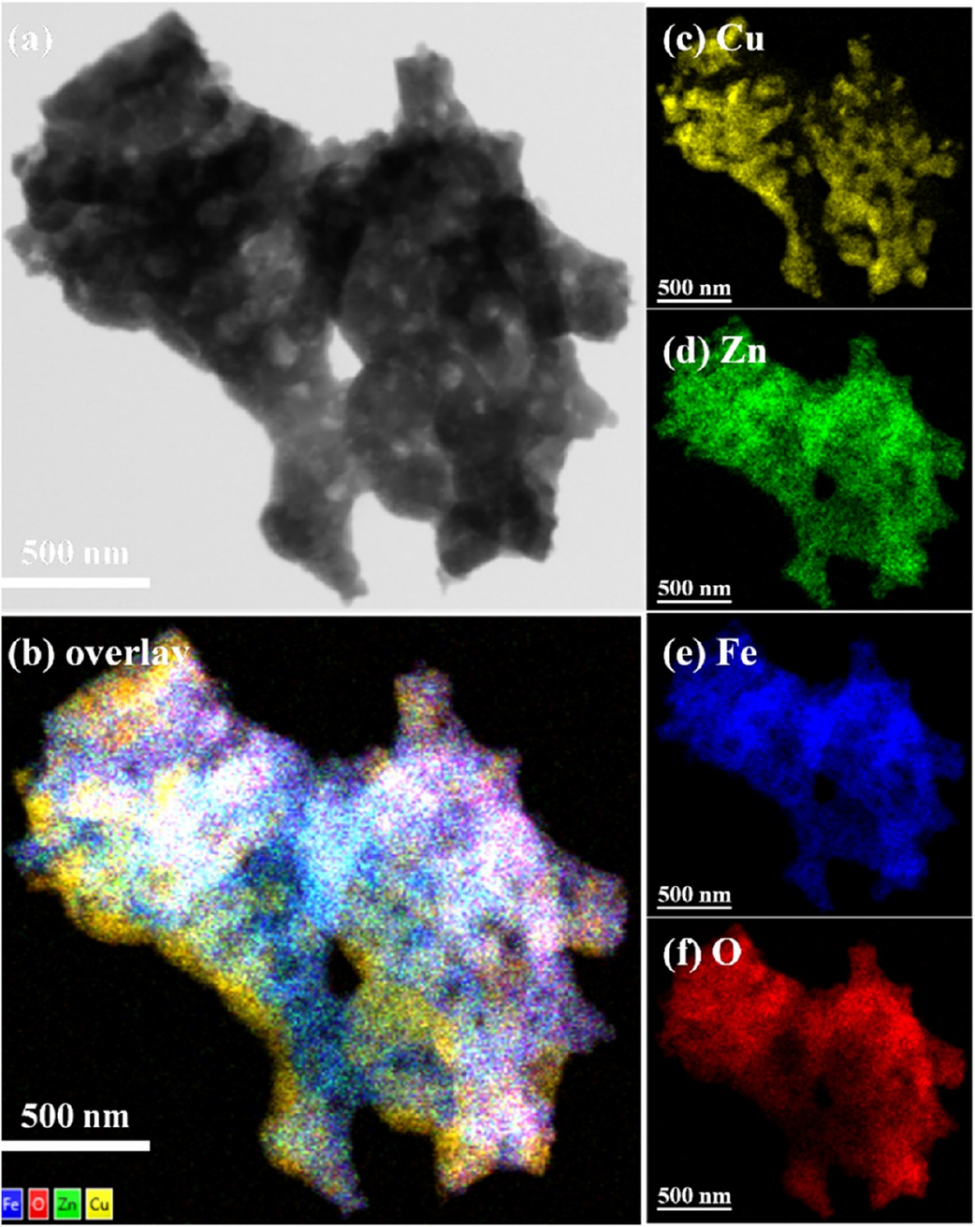
STEM-EDX mapping images of the 60CuFeO_2_–40ZnFe_2_O_4_ nanocomposite prepared by the GNP method (a) STEM image, (b) Cu, Zn, Fe and O overlap, (c) Cu, (d) Zn, (e) Fe and (f) O.

**Figure 8:**
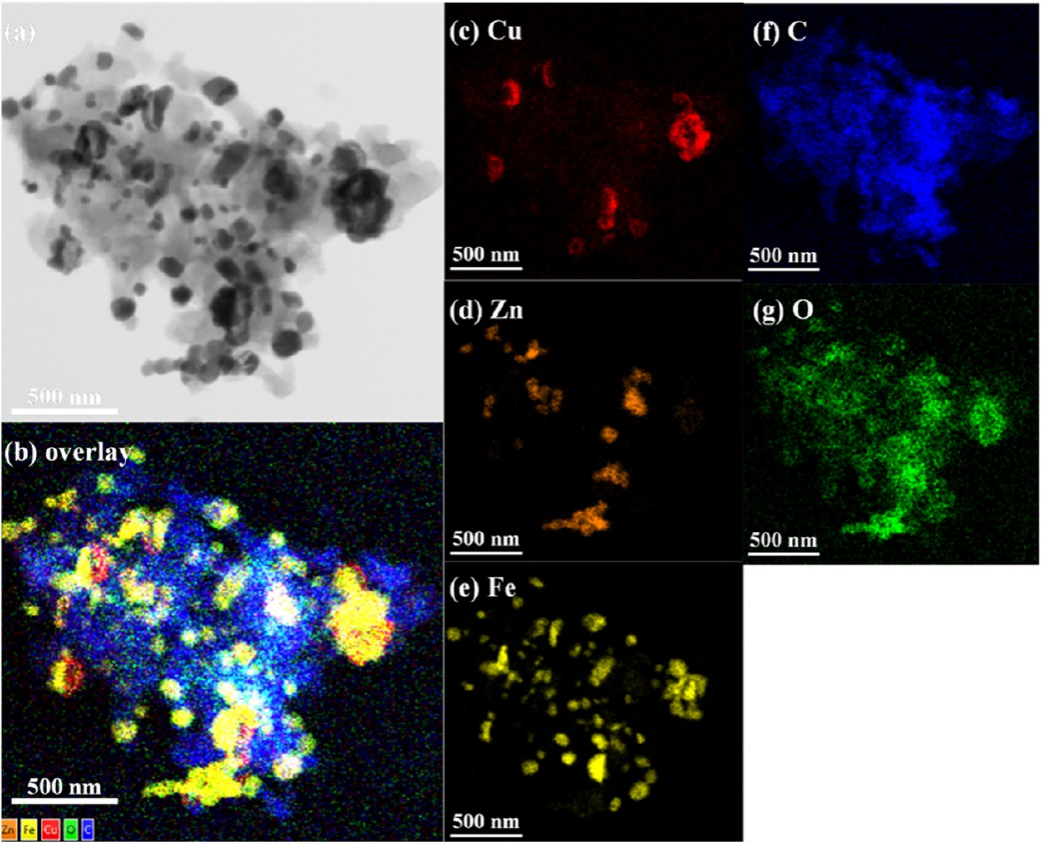
STEM-EDX mapping images of the 60CuFeO_2_–40ZnFe_2_O_4_ nanocomposite prepared by the GNP method after SRM process (a) STEM image, (b) Cu, Zn, Fe, C, and O overlap, (c) Cu, (d) Zn, (e) Fe, (f) C, and (g) O.

**Figure 9:**
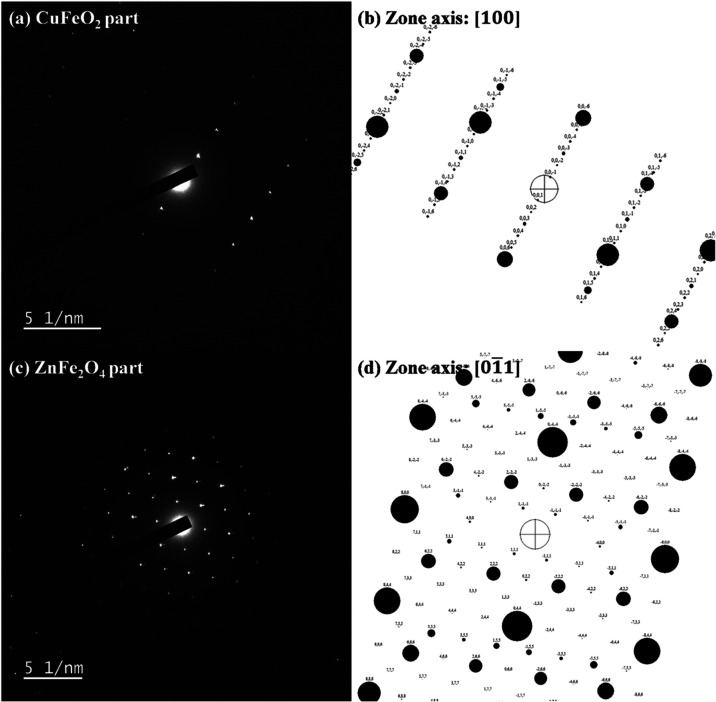
TEM-SAED diffraction patterns of the 60CuFeO_2_–40ZnFe_2_O_4_ nanocomposite prepared by the GNP method (a) CuFeO_2_ part, (b) simulated diffraction pattern (Zone axis) of CuFeO_2_, (c) ZnFe_2_O_4_ part and (d) simulated diffraction pattern (Zone axis) of ZnFe_2_O_4_.

The vibrational modes of the as-prepared nanocomposites were studied by Raman spectrum measurement under 532 nm laser irradiation. The Raman spectrum results of CuFeO_2_–ZnFe_2_O4 nanocomposites with different incorporations are shown in Figure 10. In general, the different structures exhibited different vibrational modes. Delafossite has E_g_ and A_1g_ symmetric Raman modes, and spinel oxide has five Raman vibration modes, namely, 1A_g_, 1E_g_, and 3F_2g_, respectively.^48,49^ As shown in Figure 10, two vibrational peaks at 352 and 691 cm^–1^ were observed in the CuFeO_2_ delafossite material, which corresponded to the E_g_ and A_1g_ symmetric Raman modes. The A_1g_ modes represented the vibrations of the Cu–O bonds in the *c*-axis. The E_g_ mode was attributed to the triangular lattice vibrations perpendicular to the *c*-axis. On the other hand, the vibrational peaks of ZnFe_2_O_4_ could be observed at 167, 240, 315, 460, and 630 cm^–1^, which were related to the (1) F_2g_, E_g_, (2) F_2g_, (3) F_2g_, and A_1g_ vibration modes of the ZnFe_2_O_4_ spinel structure. Furthermore, the Raman spectrum of the ZnFe_2_O_4_ nanopowder exhibited peaks at around 1090 and 1290 cm^–1^, which could be considered second-order signals from hematite.^50–52^

**Figure 10:**
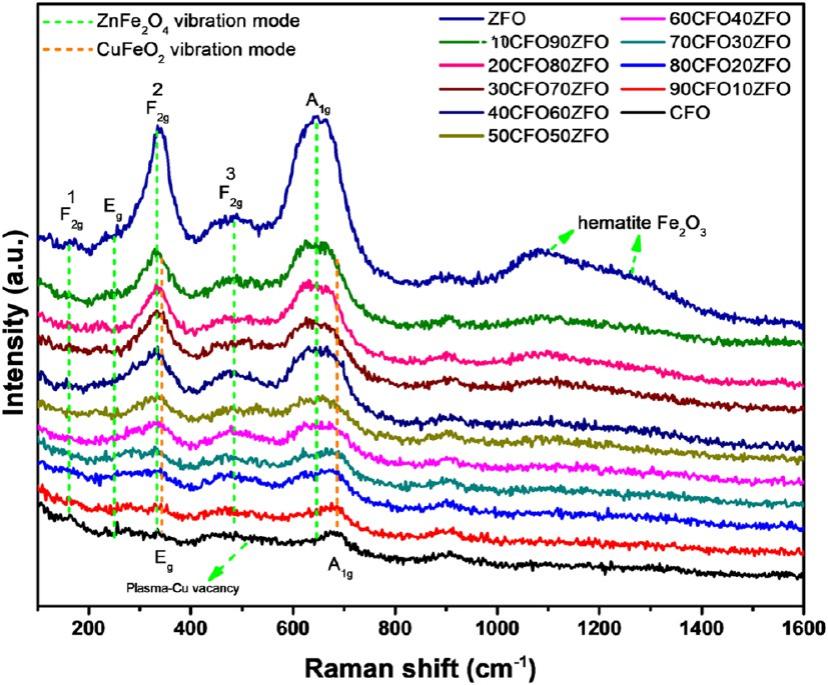
Raman analysis of CuFeO_2_–ZnFe_2_O_4_ nanocomposites prepared by the GNP method.

Normally, a catalyst can be influenced by its own effective reaction area. To investigate the influence of the porous structure and higher specific surface area (SSA) of the as-prepared nanocomposites, BET measurements were used to analyze the CuFeO_2_–ZnFe_2_O_4_ nanocomposite prepared by the GNP method to determine the SSA, and the findings are listed in Table 1. Before BET measurement processing, the specimen had to be degassed at 200 °C with high purity N_2_ (4N) gas to purge the remaining water. During the BET measurement, the adsorption volume of N_2_ to different relative pressures varied, with *P*/*P*_0_ = 0–0.3. According to Table 1, the SSA of CuFeO_2_–ZnFe_2_O_4_ nanocomposite materials with different incorporations varied from 1.90 to 6.32 m^2^/g. Compared with the surface area of CuFeO_2_ synthesized by solid-state reaction reported in a previous study (0.57 m^2^/g), the SSAs of the CuFeO_2_–ZnFe_2_O_4_ nanocomposite materials prepared by GNP were approximately 1 to 2 orders higher than that of the powder prepared via solid-state reaction. Due to the high surface area, the catalysts had larger reaction contact areas and more reaction sites, which could lead to better performance.

**Table 1:** Specific Surface Areas of CuFeO_2_–ZnFe_2_O_4_ Porous Powders Prepared by GNP

**sample conditions**	**specific surface area** **(m^2^/g)**
CuFeO_2_	4.38
90CuFeO_2_-10ZnFe_2_O_4_	3.22
80CuFeO_2_-20ZnFe_2_O_4_	3.74
70CuFeO_2_-30ZnFe_2_O_4_	4.54
60CuFeO_2_-40ZnFe_2_O_4_	2.08
50CuFeO_2_-50ZnFe_2_O_4_	3.83
40CuFeO_2_-60ZnFe_2_O_4_	2.19
30CuFeO_2_-70ZnFe_2_O_4_	1.90
20CuFeO_2_-80ZnFe_2_O_4_	2.29
10CuFeO_2_-90ZnFe_2_O_4_	2.98
ZnFe_2_O_4_	6.32
CuCrO_2_ prepared by solid state^48^	0.47
CuFeO_2_ prepared by solid-state^49^	0.57

Temperature-programmed reduction (TPR) is one method used to investigate the redox behavior of metal oxides and mixed metal oxides. Figure 11 illustrates the H_2_-TPR profiles of the CuFeO_2_–ZnFe_2_O_4_ nanocomposites from room temperature to 800 °C with a heating rate of 10 °C/min in an atmosphere of 10% H_2_/N_2_. For CuFeO_2_, three broadened peaks occurred at approximately 300, 340, and 470 °C during heating in a hydrogen atmosphere, as shown in Figure 11. The peaks located at lower temperatures corresponded to the reduction of Cu^+^ to Cu at the surface of the CuFeO_2_ nanopowder, which was triggered at 240 °C and reached its maximum redox reaction at 310 °C.^41^ The subsequent peaks appearing at 340 and 470 °C were consistent with the reduction of Fe_3_O_4_, which started at a temperature overlapping that of the former peak and maximized at 470 °C. Compared to the CuFeO_2_, the ZnFe_2_O_4_ was more difficult to reduce in a 10% H_2_/N_2_ atmosphere as the temperature increased. The broadened reduction peaks of the ZnFe_2_O_4_ nanopowder were located at approximately 535 and 595 °C in an atmosphere of 10% H_2_/N_2_..^53^ Moreover, the reduction peaks of the CuFeO_2_–ZnFe_2_O_4_ nanocomposites decreased the reduction temperature with different incorporations, which revealed the spillover phenomena occurring in these results. The reaction sequence of the reduction of CuFeO_2_ and ZnFe_2_O_4_ follows the path mentioned in eqs 17 to 22.

5.1 $$2\mathrm{CuFe}O_{2}+H_{2}\to 2{\mathrm{Cu}}_{2}O+{\mathrm{Fe}}_{2}O_{3}+2H_{2}$$

5.2 $$3{\mathrm{Cu}}_{2}O+{3\mathrm{Fe}}_{2}O_{3}+2H_{2}\to 3{\mathrm{Cu}}^{*}+{2\mathrm{Fe}}_{3}O_{4}+2H_{2}O$$

5.3 $$3{\mathrm{Cu}}^{*}+2{\mathrm{Fe}}_{3}O_{4}+8H_{2}\to 3{\mathrm{Cu}}^{*}+6{\mathrm{Fe}}^{*}+8H_{2}O$$

5.4 $$\mathrm{Zn}{\mathrm{Fe}}_{2}O_{4}+H_{2}\to \mathrm{ZnO}+{\mathrm{Fe}}_{2}O_{3}+H_{2}$$

5.5 $$\mathrm{ZnO}+3{\mathrm{Fe}}_{2}O_{3}+H_{2}\to \mathrm{ZnO}+{2\mathrm{Fe}}_{3}O_{4}+H_{2}O$$

5.6 $$\mathrm{ZnO}+{2\mathrm{Fe}}_{3}O_{4}+{8H}_{2}\to \mathrm{ZnO}+6{\mathrm{Fe}}^{*}+{8H}_{2}O$$

**Figure 11:**
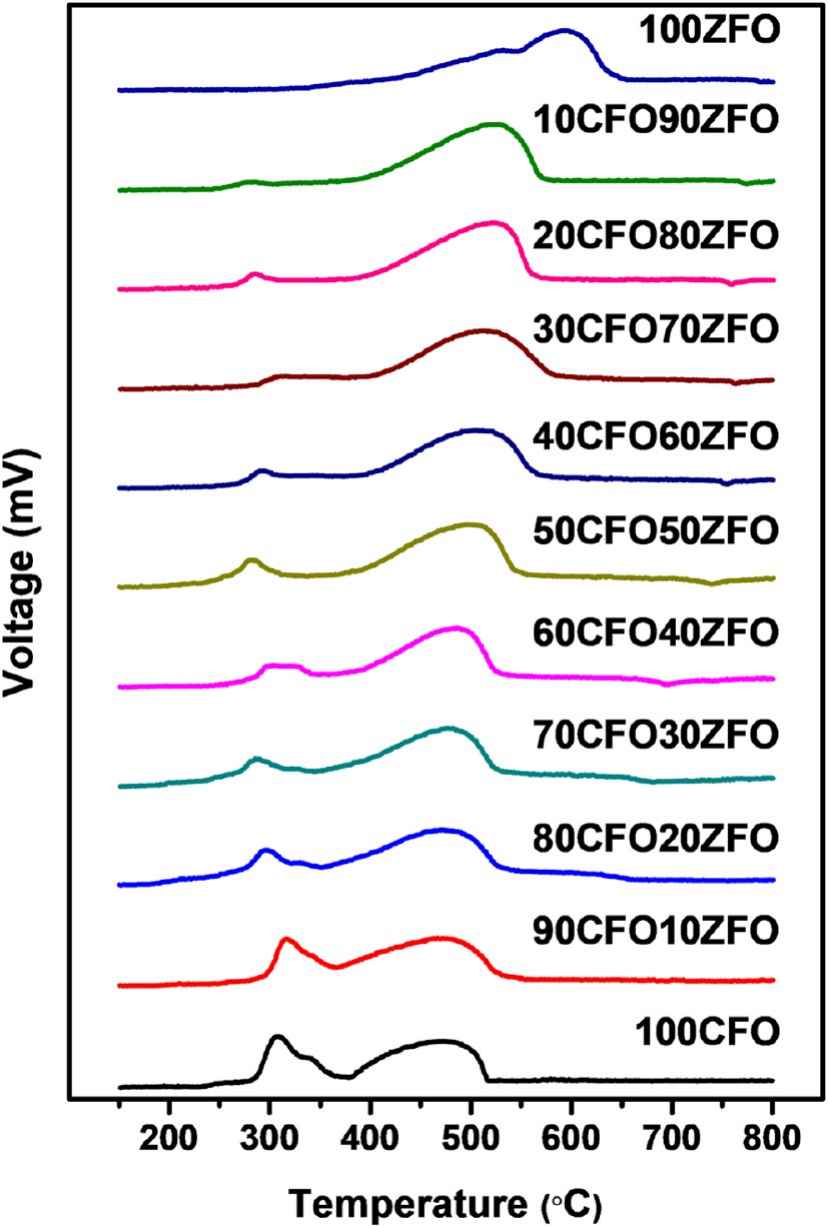
H_2_-TPR profiles of CuFeO_2_–ZnFe_2_O_4_ nanocomposites prepared by the GNP method.

The theoretical H_2_ uptake for CuFeO_2_ and ZnFe_2_O_4_ is 1 mol H_2_/151.4 g (6.61 mmol H_2_/g) and 1 mol H_2_/241.1 g (4.15 mmol H_2_/g), respectively. Theoretical hydrogen uptake was calculated based on the stoichiometric reduction of Cu^+^ to Cu^0^ and Fe^3+^ to Fe^2+^ in CuFeO_2_, and Fe^3+^ to Fe^2+^ in ZnFe_2_O_4_.^38,48^ The experimental hydrogen consumption for CuFeO_2_ and ZnFe_2_O_4_ was determined to be 6.31 and 3.88 mmol/g, respectively, corresponding to 95.5 and 93.5% of the calculated theoretical uptake. These high reduction efficiencies indicate that the majority of redox-active Cu^+^ and Fe^3+^ species are readily accessible and effectively participate in the reduction process. The close agreement between theoretical and experimental values suggests that the GNP synthesis resulted in highly reducible, structurally open nanostructures with minimal diffusion limitations. This behavior reinforces the proposed synergistic mechanism between the Cu and Zn components, where Cu facilitates methanol activation via Cu^+^/Cu^0^ cycling, and ZnFe_2_O_4_ enhances oxygen mobility and structural stability. The redox efficiency achieved here is notably higher, highlighting the strong potential of the CuFeO_2_–ZnFe_2_O_4_ nanocomposite for methanol steam reforming applications. These findings provide direct mechanistic evidence supporting the high redox efficiency and practical applicability of the CuFeO_2_–ZnFe_2_O_4_ nanocomposite catalyst system for efficient hydrogen production.

X-ray photoelectron spectroscopy (XPS) was used to investigate the chemical composition status and the binding valence state with each element of the CuFeO_2_–ZnFe_2_O_4_ nanocomposite. Figure 12a–d present the Cu 2p, Zn 2p, Fe 2p, and O 1s XPS spectra and the positions of the deconvolution peaks of the CuFeO_2_–ZnFe_2_O_4_ nanocomposites. As depicted in Figure 12a, Cu 2p spectra of the as-combusted Cu-based nanopowder and nanocomposite (CuFeO_2_ and 60CuFeO_2_–40ZnFe_2_O_4_) revealed the satellite peaks, which consisted of the amorphous CuO (Cu (II)) and CuFeO_2_(Cu (I)) existing in the Cu-based nanopowder. Moreover, the Cu 2p spectra of 60CuFeO_2_–40ZnFe_2_O_4_ nanocomposite and CuFeO_2_ revealed that Cu 2p had two main peaks, located at approximately 934.3 and 954.1 eV, which were related to Cu 2p_3/2_ and Cu 2p_1/2_, respectively.^54^ After the SRM process, the 60CuFeO_2_–40ZnFe_2_O_4_ nanocomposite exhibited peaks at 934.3 and 954.1 eV, which were related to Cu 2p_3/2_ and Cu 2p_1/2_, respectively. However, the XPS spectrum intensity decreased due to the decrease in the relative content of Cu atoms, and the Cu atoms diffused toward the bulk. Furthermore, after the SRM process, the Cu^1+^ was reduced to Cu^0^, which was comparable to the XRD results.

**Figure 12:**
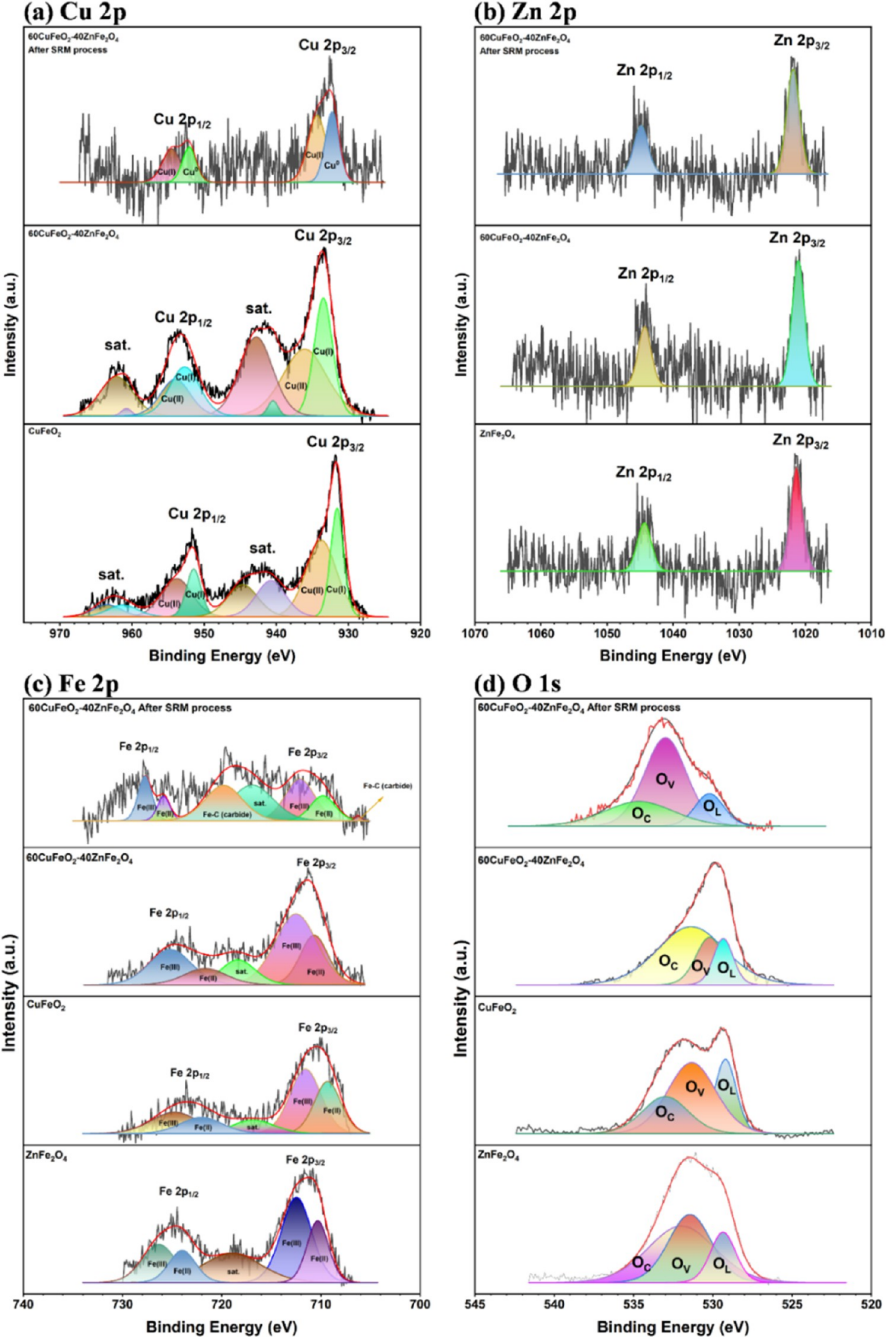
XPS spectra of CuFeO_2_–ZnFe_2_O_4_ nanocomposite prepared by GNP (a) Cu 2p, (b) Zn 2p, (a) Fe 2p, and (d) O 1s.

As illustrated in Figure 12b, the CuFeO_2_–ZnFe_2_O_4_ nanocomposites showed two main peaks of 1044.1 and 1021.2 eV before and after the SRM process, which were related to Zn 2p_1/2_ and Zn 2p_3/2_ of the Zn^2+^, respectively. According to Figure 12c, the Fe 2p spectra of as-combusted GNP powder (ZnFe_2_O_4_, CuFeO_2_, and 60CuFeO_2_–40ZnFe_2_O_4_) exhibited two central peaks and a satellite peak at 711.0, 724.5, and 718.1–719.5 eV, corresponding to Fe 2p_3/2_ and Fe 2p_1/2_ and a satellite, respectively.^19^ During the SRM process, carbides were formed from the Fe and C from the coke, CO_2_, or CO, as can observed in the Fe 2p spectra of the 60CuFeO_2_–40ZnFe_2_O_4_ nanocomposite after the SRM process.

Figure 12d reveals the O 1s spectra of the 60CuFeO_2_–40ZnFe_2_O_4_ nanocomposite, which consisted of three peaks in this energy region, including chemisorbed oxygen species (O_C_), oxygen vacancy (O_V_), and lattice oxygen (O_L_).^55^ According to Figure 12d, the as-combusted nanocomposite showed a higher intensity of O_C_ than that of the 60CuFeO_2_–40ZnFe_2_O_4_ nanocomposite after the SRM process, as the porous structure of the as-combusted nanocomposite could effectively adsorb oxygen on the surface. On the other hand, the 60CuFeO_2_–40ZnFe_2_O_4_ nanocomposite, after the SRM process, was covered with carbon and formed carbides on the surface of the nanocomposite. The peaks of O_V_ and O_L_ could be related to oxygen vacancies and the crystallinity of the material; the O_L_ peak’s intensity of the 60CuFeO_2_–40ZnFe_2_O_4_ was lower than those of ZnFe_2_O_4_ and CuFeO_2_, which could be attributed to the material, after compounding, inhibiting the crystallinity; after the SRM process, the 60CuFeO_2_–40ZnFe_2_O_4_ nanocomposite exhibited higher intensity of the O_V_, which could be considered to indicate decomposition of the 60CuFeO_2_–40ZnFe_2_O_4_ nanocomposite into carbides, ZnO, and Cu.

The H_2_ production performance of the CuFeO_2_–ZnFe_2_O_4_ nanocomposite was evaluated with a gas chromatograph equipped with a TCD detector in the SRM process. The SRM process reaction temperature varied between 350 and 550 °C, and N_2_ was used as the carrier gas, with a flow rate of 30 sccm­(mL/min). The H_2_ production rate was transformed into an output rate of milliliters per minute per gram-catalyst (mL STP min^–1^ g-cat^–1^). In addition, the SRM process was performed with the CuFeO_2_, 90CuFeO_2_-10ZnFe_2_O_4_, 80CuFeO_2_-20ZnFe_2_O_4_, 70CuFeO_2_-30ZnFe_2_O_4_, 60CuFeO_2_-40ZnFe_2_O_4_, 50CuFeO_2_-50ZnFe_2_O_4_, 40CuFeO_2_-60ZnFe_2_O_4_, 30CuFeO_2_-70ZnFe_2_O_4_, 20CuFeO_2_-80ZnFe_2_O_4_, 10CuFeO_2_-90ZnFe_2_O_4_, and ZnFe_2_O_4_ nanocomposites, as shown in Table 2 and Figure 13, respectively. It can be observed that, as the reaction temperature increased, the hydrogen production performance of the catalyst exhibited a gradual enhancement up to 500 °C. Among the CuFeO_2_–ZnFe_2_O_4_ nanocomposite series, the 60CuFeO_2_–40ZnFe_2_O_4_ nanocomposite exhibited the best H_2_ production performance at 500 °C, reaching 6984.7 mL STP min^–1^ g-cat^–1^ (or) 312 ± 2 mmol STP min^–1^ g-cat^–1^. In addition, the 70CuFeO_2_-30ZnFe_2_O_4_ and 50CuFeO_2_-50ZnFe_2_O_4_ nanocomposites also revealed higher H_2_ production rates at 450 and 500 °C, which corresponded to the H_2_-TPR profile of the CuFeO_2_–ZnFe_2_O_4_ nanocomposite. Although higher specific surface area (SSA) is generally associated with enhanced catalytic performance, the correlation between BET of SSA and H_2_ production rate in Table 1 is not strictly monotonic. For example, ZnFe_2_O_4_ exhibited the highest surface area (6.32 m^2^/g) but showed only moderate hydrogen production compared to 60CuFeO_2_–40ZnFe_2_O_4_, which had a lower surface area (2.08 m^2^/g) but the highest H_2_ output. This discrepancy can be attributed to the dominant influence of active site composition and redox properties rather than surface area alone. In this system, the synergistic catalytic behavior of Cu and Zn components, particularly the Cu^+^/Cu^0^ redox cycle and the oxygen storage capacity of ZnFe_2_O_4_, plays a more critical role in SRM efficiency than total surface area. Additionally, the distribution and accessibility of active sites (which are not always directly represented by BET SSA) likely contribute to the overall catalytic activity. Therefore, while SSA provides useful information about porosity, it is not the sole determining factor for hydrogen productivity in this nanocomposite system. Based on the results of the H_2_ catalytic test, the hydrogen generation performance improved when a small amount of Zn-based material was incorporated. Moreover, the hydrogen generation performance rate of the CuFeO_2_–ZnFe_2_O_4_ nanocomposites was related to the two materials (CuFeO_2_ and ZnFe_2_O_4_) in the nanocomposites and had a synergistic effect, as can be observed in Table 2 and Figure 13. Therefore, CuFeO_2_–ZnFe_2_O_4_ nanocomposites could be directly applied as the catalyst of the SRM process without the activation process.

**Figure 13:**
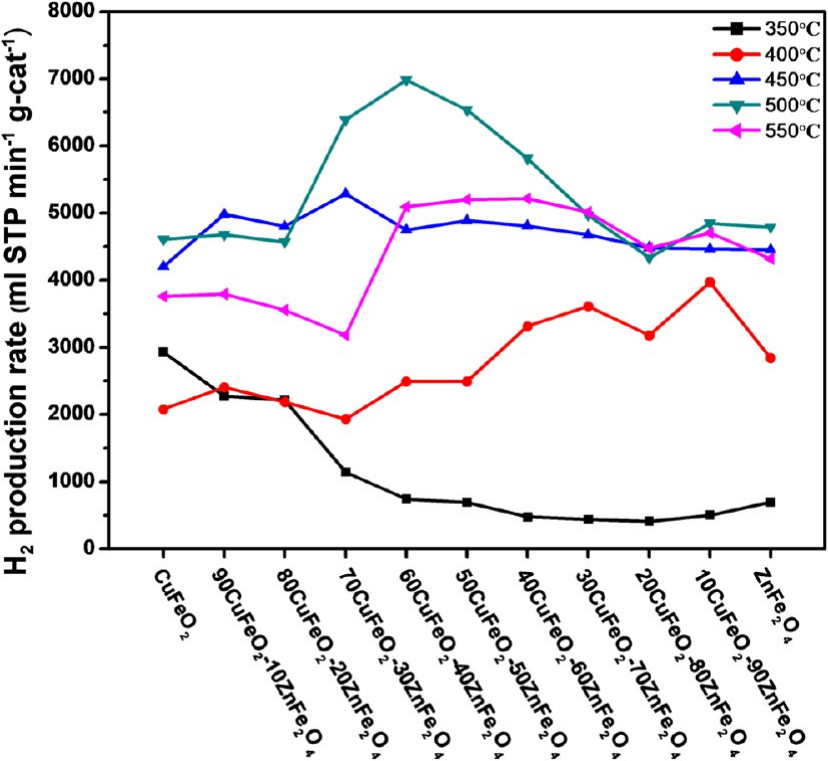
Plots of CuFeO_2_–ZnFe_2_O_4_ nanocomposite H_2_ production rates in the SRM process at 350–-550 °C.

**Table 2:** Rates of the CuFeO_2_–ZnFe_2_O_4_ Nanocomposite H_2_ Production at 350–550°C at an N_2_ Flow Rate of 30 sccm (mL/min)

	**rate of H**_**2**_ **production (mL STP min** ^**–1**^ **g-cat^–^** ^**1**^ **)**				
**samples**	**350 °C**	**400 °C**	**450 °C**	**500 °C**	**550 °C**
CuFeO_2_	2927 ± 84	2075 ± 71	4201 ± 49	4611 ± 44	3760 ± 67
90CuFeO_2_-10ZnFe_2_O_4_	2275 ± 51	2406 ± 56	4985 ± 45	4676 ± 41	3793 ± 11
80CuFeO_2_-20ZnFe_2_O_4_	2218 ± 18	2186 ± 51	4804 ± 27	4568 ± 68	3558 ± 54
70CuFeO_2_-30ZnFe_2_O_4_	1141 ± 38	1934 ± 52	5285 ± 15	6389 ± 60	3177 ± 25
60CuFeO_2_–40ZnFe_2_O_4_	741 ± 14	2491 ± 14	4747 ± 43	6984 ± 35	5090 ± 44
50CuFeO_2_-50ZnFe_2_O_4_	691 ± 14	2491 ± 23	4891 ± 83	6538 ± 57	5202 ± 98
40CuFeO_2_-60ZnFe_2_O_4_	473 ± 18	3311 ± 15	4806 ± 12	5817 ± 18	5219 ± 60
30CuFeO_2_-70ZnFe_2_O_4_	436 ± 28	3613 ± 22	4673 ± 36	4971 ± 41	5014 ± 15
20CuFeO_2_-80ZnFe_2_O_4_	410 ± 12	3180 ± 19	4482 ± 32	4334 ± 83	4479 ± 37
10CuFeO_2_-90ZnFe_2_O_4_	502 ± 24	3966 ± 72	4463 ± 44	4850 ± 18	4704 ± 48
ZnFe_2_O_4_	696 ± 14	2840 ± 37	4451 ± 34	4791 ± 21	4321 ± 81

Generally, SRM is related to a primary reaction ([Disp-formula eq23], SRM process) and some secondary reactions, where other secondary reactions also occur simultaneously, including ([Disp-formula eq24]) methanol decomposition, ([Disp-formula eq25]) water gas shift (WGS), ([Disp-formula eq26]) methanation of CO (MCO), and ([Disp-formula eq27]) methanation of CO_2_ (MCO_2_). The reaction formulas are as follows.

6.1 $${\mathrm{CH}}_{3}\mathrm{OH}+H_{2}O⇄{\mathrm{CO}}_{2}+3H_{2}$$

6.2 $${\mathrm{CH}}_{3}\mathrm{OH}⇄\mathrm{CO}+2H_{2}$$

6.3 $$\mathrm{CO}+H_{2}O⇄{\mathrm{CO}}_{2}+H_{2}$$

6.4 $$\mathrm{CO}+3H_{2}⇄{\mathrm{CH}}_{4}+H_{2}O$$

6.5 $${\mathrm{CO}}_{2}+4H_{2}⇄{\mathrm{CH}}_{4}+2H_{2}O$$

Figure 14 shows the selectivity and conversion results of the 60CuFeO_2_–40ZnFe_2_O_4_ nanocomposite at 350 to 550 °C, including Figure 14a H_2_, Figure 14b CH_4_, Figure 14c CO, and Figure 14d CO_2_. Based on Figure 14a, the 60CuFeO_2_–40ZnFe_2_O_4_ nanocomposite exhibited higher H_2_ selectivity, exceeding 98.7% in each reaction temperature condition, and the H_2_ conversion efficiency of the 60CuFeO_2_–40ZnFe_2_O_4_ nanocomposite also maintained over 90% conversion efficiency and had a slightly increasing trend with the temperature increase from 350 to 400 °C, which could be considered as the water gas shift in progress. Furthermore, CH_4_ appeared due to the methanation reaction of CO and CO_2_ (MCO and MCO_2_) during the process.^45^ According to Figure 14c,d, the increase in the amount of CO with the rise in reaction temperature occurred due to the reverse water gas shift (rWGS) occurring and producing the CO.

**Figure 14:**
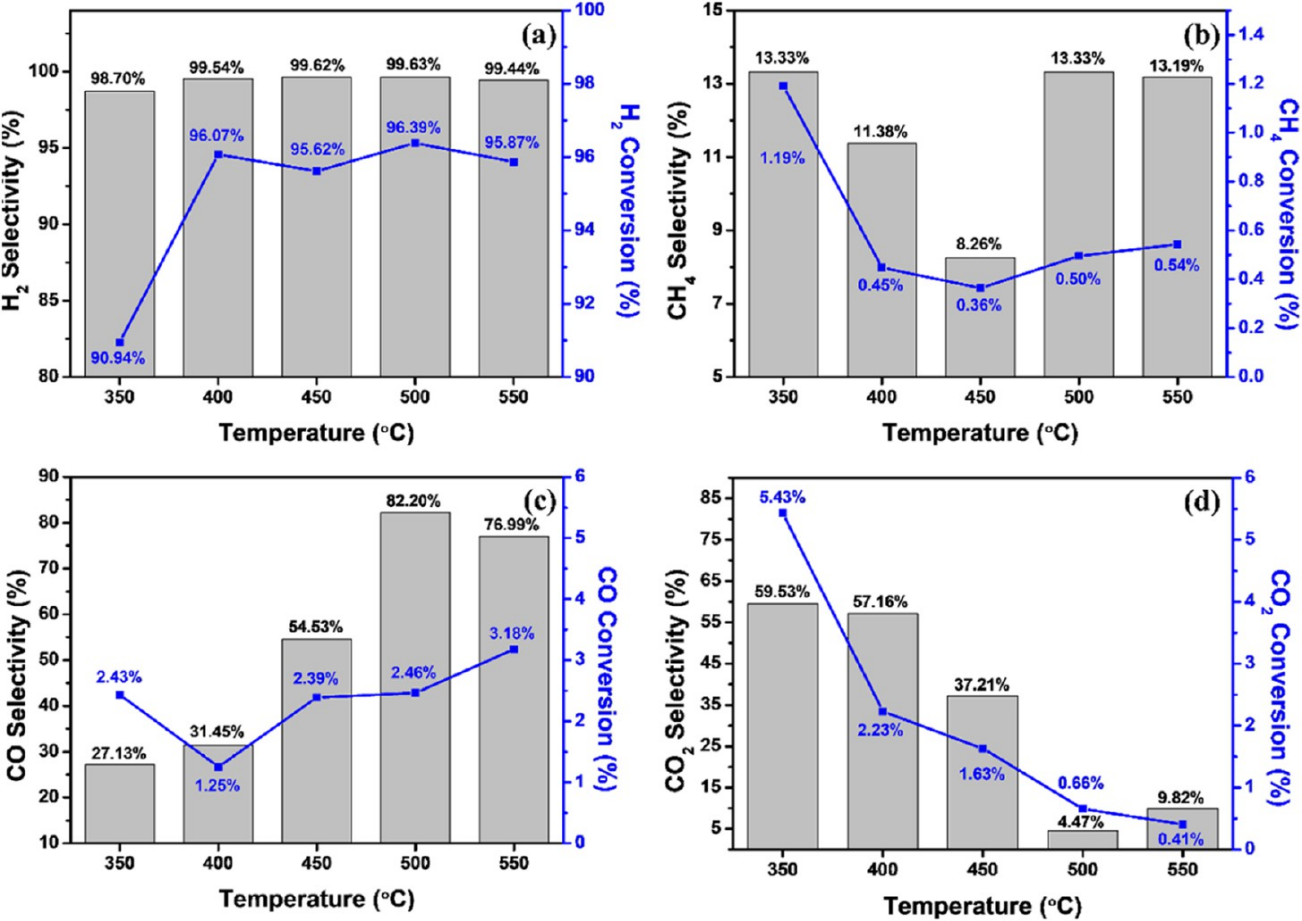
Selectivity/conversion columns and plots of 60CuFeO_2_–40ZnFe_2_O_4_ nanocomposite H_2_ production rates in the SRM process at 350–550 °C (a) H_2_, (b) CH_4_, (c) CO and (d) CO_2_.

Other crucial aspects of catalyst performance are the stability, cycle-life, and reusability of the material. In this study, the 60CuFeO_2_–40ZnFe_2_O_4_ nanocomposite was examined for its stability and reusability, with a focus on the cycle-life assessment, as illustrated in Figure 15. The 60CuFeO_2_–40ZnFe_2_O_4_ nanocomposite was employed in three consecutive SRM processes at 500 °C with a N_2_ flow rate of 30 sccm­(mL/min). As illustrated in Figure 15, the hydrogen production rate of the 60CuFeO_2_–40ZnFe_2_O_4_ nanocomposite in a 500 °C SRM process exhibited minimal variation following three SRM process testing cycles. The rate of H_2_ harvesting decreased by approximately 3% only, resulting in the 60CuFeO_2_–40ZnFe_2_O_4_ nanocomposite exhibiting excellent stability and reusability.

**Figure 15:**
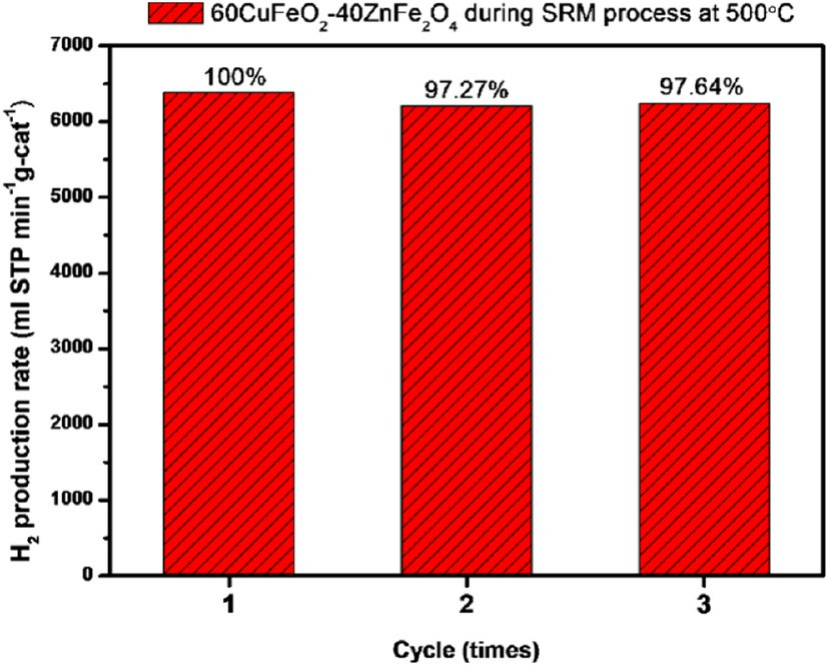
Cycle-life examination of the 60CuFeO_2_–40ZnFe_2_O_4_ nanocomposite during SRM at 500 °C with an N_2_ flow rate of 30 sccm (mL/min).

To facilitate a comparison with previous works in the field of SRM research with regard to the catalytic properties, Table 3 presents the H_2_ production and stability performances of each catalyst from previous SRM studies.^19,29,32,42,56–59^ In the aspect of H_2_ production performance, 60CuFeO_2_–40ZnFe_2_O_4_ nanocomposite had higher H_2_ production efficiency and stability than the catalysts in the aforementioned studies. Moreover, the 60CuFeO_2_–40ZnFe_2_O_4_ nanocomposite exhibited highly stable properties relative to those of other catalysts and commercial products. Based on the SRM process conducted in this study, the 60CuFeO_2_–40ZnFe_2_O_4_ nanocomposite has a high H_2_ production performance and good stability at the same time. Consequently, this CuFeO_2_–ZnFe_2_O_4_ nanocomposite could be employed as a prospective material for H_2_ generation, which could effectively circumvent the perils associated with the direct utilization of hydrogen, including the potential for combustion and explosion, and applied to vehicle engines and fuel cells.

**Table 3:** Comparison of H_2_ Production Performance and Stability with Previous SRM Research and Commercial Catalysts

**catalyst condition**	**temperature (°C)**	**rate of H**_**2**_ **production (mL STP min** ^–**1**^ **g-cat** ^–**1**^ **)**	**stability** (times/performance)
60CuFeO_2_–40ZnFe_2_O_4_ (this work)	**500**	**6985**	**3 times/97%**
ZnCr_0.4_Fe_1.6_O_4_ (GNP)^19^	450	6850	3 times/60%
			4 times/47%
4 times/47% ZnCr_2_O_4_ (GNP)^42^	450	3549	3 times/10%
CuFe_2_O_4_ (bulk)^56^	400	277	
CuYO_2_ (GNP)^57^	300	1735	
CuFeO_2_ (GNP)^32^	400	2010	
CuFeO_2_–CeO_2_ (GNP)^32^	400	2582	
Cu/Zn/Al (commercial catalyst)^58^	400	263	
CuCrO_2_ (GNP)^29^	400	1720	
CuCrO_2_ (bulk)^59^	340	240	

## 4 Conclusions

A series of CuFeO_2_–ZnFe_2_O_4_ porous nanocomposite catalysts were synthesized via the glycine nitrate process (GNP), exhibiting high surface areas and coral-like porous morphology. Among them, the 60CuFeO_2_–40ZnFe_2_O_4_ composition demonstrated the highest hydrogen production rate of 6984 ± 35 mL STP min^–1^ g-cat^–1^ at 500 °C, without activation treatment and retained 97% performance after three cycles, confirming excellent stability. This enhanced catalytic activity is attributed to the synergistic interaction between CuFeO_2_ and ZnFe_2_O_4_ phases and the increased surface area (up to 6.32 m^2^/g) achieved via GNP. These findings suggest that CuFeO_2_–ZnFe_2_O_4_ nanocomposites are promising low-cost and efficient catalysts for methanol steam reforming (SRM)-based hydrogen production. Future work should explore catalyst integration in compact reforming systems, long-term operational stability, and performance under realistic process conditions. Additionally, coupling these materials with membrane reactors or fuel cell systems may offer a pathway toward safer and scalable on-demand hydrogen generation.

## References

[1] Ellabban O., Abu-Rub H., Blaabjerg F. (2014). Renewable energy resources: Current
status, future prospects, and their enabling technology. Renewable Sustainable Energy Rev..

[2] Hu B., Shu R., Tian Z., Wang C., Chen Y., Xu Y. (2024). Enhancement
of hydrogen production via methanol steam reforming using a Ni-based
catalyst supported by spongy mesoporous alumina. Green Chem..

[3] Vinoth S., Pandikumar A. (2024). Recent advances
in bismuth oxyfluoride-based photocatalysts
for energy and environmental remediation. Mater.
Today Chem..

[4] Vinoth S., Ong W. J., Pandikumar A. (2022). Defect engineering
of BiO_X_ (X = Cl, Br, I) based photocatalysts for energy
and environmental
applications: Current progress and future perspectives. Coord. Chem. Rev..

[5] Schorn F., Breuer J. L., Samsun R. C., Schnorbus T., Heuser B., Peters R., Stolten D. (2021). Methanol as a renewable
energy carrier: An assessment of production and transportation costs
for selected global locations. Adv. Appl. Energy..

[6] Lindström B., Pettersson L. J., Govind M. P. (2002). Activity and characterization of
Cu/Zn, Cu/Cr, and Cu/Zr on γ-alumina for methanol reforming
for fuel cell vehicles. Appl. Catal., A.

[7] Armaroli N., Balzani V. (2007). The future of energy
supply: Challenges and opportunities. Angew.
Chem., Int. Ed..

[8] Lee J. E., Shafiq I., Hussain M., Lam S. S., Rhee G. H., Park Y. K. (2022). A review on integrated thermochemical hydrogen production
from water. Int. J. Hydrogen Energy.

[9] Kannan N., Vakeesan D. (2016). Solar energy for future
world: A review. Renewable Sustainable Energy
Rev..

[10] Zhou Y., Yu L., Chang J., Feng L., Zhang J. (2024). Low carbon alcohol
fuel electrolysis of hydrogen generation catalyzed by a novel and
effective Pt–CoTe/C bifunctional catalyst system. Green Energy Environ..

[11] Pal D. B., Chand R., Upadhyay S. N., Mishra P. K. (2018). Performance
of water
gas shift reaction catalysts: A review. Renewable
Sustainable Energy Rev..

[12] Di
Nardo A., Portarapillo M., Russo D., Di B. A. (2024). Hydrogen
production via steam reforming of different fuels: thermodynamic comparison. Int. J. Hydrogen Energy.

[13] Bepari S., Kuila D. (2020). Steam reforming of methanol, ethanol and glycerol over nickel-based
catalysts-A review. Int. J. Hydrogen Energy.

[14] Palo D. R., Dagle R. A., Holladay J. D. (2007). Methanol
steam reforming for hydrogen
production. Chem. Rev..

[15] Iulianelli A., Ribeirinha P., Mendes A., Basile A. (2014). Methanol steam reforming
for hydrogen generation via conventional and membrane reactors: A
review. Renewable Sustainable Energy Rev..

[16] Darsan A. S., Pandikumar A. (2024). Recent research
progress on metal halide perovskite-based
visible light active photoanode for photoelectrochemical water splitting. Mater. Sci. Semicond. Process..

[17] Ganesh V., Pandikumar A., Alizadeh M., Kalidoss R., Baskar K. (2020). Rational design
and fabrication of surface tailored low dimensional Indium Gallium
Nitride for photoelectrochemical water cleavage. Int. J. Hydrogen Energy.

[18] Shokrani R., Haghighia M., Ajameina H., Abdollahifara M. (2018). Hybrid sonochemic
urea-nitrate combustion preparation of CuO/ZnO/Al_2_O_3_ nanocatalyst used in fuel cell-grade hydrogen production
from methanol: Effect of sonication and fuel/nitrate ratio. Part. Sci. Technol..

[19] Wu T. H., Yu C. L., Chen J. H., Huang J. R., Sakthinathan S., Kameoka S., Chiu T. W., Lin C. C., Fan L., Lee Y. H., Chen P. C. (2024). ZnCr_X_Fe_2‑X_O_4_ (X = 0–2) porous
powder prepared through self-combustion
glycine nitrate process and applied to methyl alcohol steam reforming
for production of pure hydrogen. Int. J. Hydrogen
Energy.

[20] Takezawa N., Iwasa N. (1997). Steam reforming and dehydrogenation of methanol: Difference in the
catalytic functions of copper and group VIII metals. Catal. Today.

[21] Breen J. P., Ross J. R. H. (1999). Methanol reforming for fuel-cell
applications: Development
of zirconia-containing Cu-Zn-Al catalysts. Catal.
Today.

[22] Amphlett J. C., Evans M. J., Mann R. F., Weir R. D. (1985). Hydrogen production
by the catalytic steam reforming of methanol: Part 2: Kinetics of
methanol decomposition using girdler G66B catalyst. Can. J. Chem. Eng..

[23] Shishido T., Yamamoto Y., Morioka H., Takehira K. (2007). Production of hydrogen
from methanol over Cu/ZnO and Cu/ZnO/Al_2_O_3_ catalysts
prepared by homogeneous precipitation: Steam reforming and oxidative
steam reforming. J. Mol. Catal. A:Chem..

[24] Liu X., Ma J., Mao L., Xu J., Xu X., Fang X., Wang X. (2024). Cu/REO (RE = La, Pr,
Sm, Y, Ce) catalysts for methanol steam reforming:
Understanding the interaction between Cu and individual REO supports. Fuel.

[25] Chen W. H., Cheng C. Y., Chih Y. K., Chein R. Y., Ubando A. T., Tabatabaei M., Lam S. S., Lin H. P. (2024). Sustainable cement
and clay support in Ni–Cu/Al_2_O_3_ catalysts
for enhancing hydrogen production from methanol steam reforming. Int. J. Hydrogen Energy.

[26] Hou X., Qing S., Liu Y., Zhang L., Gao Z. (2024). Cu-Al spinel
oxide as a sustained release catalyst for methanol steam reforming:
Enhancing the catalytic performance via surface reconstruction. J. Fuel Chem. Technol..

[27] Li C., Yao X., Zhang R., Zheng H. X., Yuan S., Yu X., Li B., Zhu M., Tu S. T. (2024). Structured nano
porous Cu/ZnO catalysts
for on-board methanol steam reforming prepared by laser powder bed
fusion and dealloying. J. Chem. Eng..

[28] Ying L. A., Liu J., Mo L., Lou H., Zheng X. (2012). Hydrogen production
by oxidative steam reforming of methanol over Ce_1‑x_Zn_x_O_y_ catalysts prepared by combustion method. Int. J. Hydrogen Energy.

[29] Chiu T. W., Hong R. T., Yu B. S., Huang Y. H., Kameoka S., Tsai A. P. (2014). Improving steam-reforming
performance by nanopowdering
CuCrO_2_. Int. J. Hydrogen Energy.

[30] Tang C. W., Chen Y. J., Yeh C. T., Wu R. C., Wang C. C., Wang C. B. (2021). Reforming of methanol
to produce hydrogen over the
Au/ZnO catalyst. Int. J. Hydrogen Energy.

[31] Shanmugam V., Neuberg S., Zapf R., Pennemann H., Kolb G. (2020). Hydrogen production over highly active
Pt-based catalyst coatings
by steam reforming of methanol: Effect of support and co-support. Int. J. Hydrogen Energy.

[32] Yu C. L., Sakthinathan S., Hwang B. Y., Lin S. Y., Chiu T. W., Yu B. S., Fan Y. J., Chuang C. (2020). CuFeO_2_-CeO_2_ nanopowder catalyst prepared by self-combustion glycine nitrate
process and applied for hydrogen production from methanol steam reforming. Int. J. Hydrogen Energy.

[33] Zhang Y., Li L., Su H., Huang W., Dong X. (2015). Binary metal oxide:
advanced energy storage materials in supercapacitors. J. Mater. Chem. A.

[34] Azad A. K., Abdalla A. M., Kumarasinghe P. I. I., Nourean S., Azad A. T., Ma J., Jiang C., Mohamed M., Dawood K., Wei B., Patabendige C. N. K. (2024). Developments and key challenges in micro/nanostructured
binary transition metal oxides for lithium-ion battery anodes. J. Energy Storage.

[35] Li S., Wu W., Yan X. (2023). Structural
and performance regulation of binary transition-metal
oxides toward supercapacitors: Advances and prospects. Renewables.

[36] Bera P. (2019). Solution Combustion
Synthesis as a Novel Route to Preparation of Catalysts. Int. J. Self-Propag. High-Temp. Synth..

[37] Merzhanov A. G. (2003). Combustion
and explosion processes in physical chemistry and technology of inorganic
materials. Russ. Chem. Rev..

[38] Miranda E. A. C., Carvajal J. F., Ma O. J. R. (2015). Effect
of the fuels glycine, urea
and citric acid on synthesis of the ceramic pigment ZnCr_2_O_4_ by solution combustion. Mater.
Res..

[39] Gao S. Y., Li H. D., Yuan J. J., Li Y. A., Yang X. X., Liu J. W. (2010). ZnO nanorods/plates
on Si substrate grown by low-temperature
hydrothermal reaction. Appl. Surf. Sci..

[40] Chao T. C., Chiu T. W., Fu Y. (2018). Fabrication
and characteristic of
delafossite-type CuFeO_2_ nanofibers by electrospinning method. Ceram. Int..

[41] Hwang B. Y., Sakthinathan S., Chiu T. W. (2019). Production of hydrogen from steam
reforming of methanol carried out by self-combusted CuCr_1‑x_Fe_x_O_2_ (x = 0–1) nanopowders catalyst. Int. J. Hydrogen Energy.

[42] Yu C. L., Sakthinathan S., Lai G. T., Lin C. C., Chiu T. W., Liu M. C. (2022). ZnO-ZnCr_2_O_4_ composite prepared
by a glycine nitrate process method and applied for hydrogen production
by steam reforming of methanol. RSC Adv..

[43] Faramawy A. M., Sayed H. M. E. (2024). Enhancement of
magnetization and optical properties
of CuFe_2_O_4_/ZnFe_2_O_4_ core/shell
nanostructure. Sci. Rep..

[44] Al
Turkestani M. K. (2024). Enhancing the photoelectrochemical performance of a
superlattice p–n heterojunction CuFe_2_O_4_/ZnFe_2_O_4_ electrode for hydrogen production. Condens. Matter.

[45] Wu Y., Zeng S., Dong Y., Fu Y., Sun H., Yin S., Guo X., Qin W. (2018). Hydrogen production
from methanol
aqueous solution by ZnO/Zn­(OH)_2_ macrostructure photocatalysts. RSC Adv..

[46] Tahay P., Khani Y., Jabari M. (2020). Synthesis of cubic and
hexagonal ZnTiO_3_ as catalyst support in steam reforming
of methanol: Study of physical and chemical properties of copper catalysts
on the H_2_ and CO selectivity and coke formation. Int. J. Hydrogen Energy.

[47] Hsu B. Z., Yu C. L., Sakthinathan S., Chiu T. W., Yu B. S., Lin C. C., Fan L., Lee Y. H. (2023). ZnO–ZnFe_2_O_4_ Catalyst for
Hydrogen Production from Methanol
Steam Reforming. Catalysts..

[48] Abrari M., Ghanaatshoar M., Malvajerdi S. S., Gholamhosseini S., Hosseini A., Sun H., Mohseni S. M. (2023). Investigating various
metal contacts for p-type delafossite α-CuGaO_2_ to
fabricate ultraviolet photodetector. Sci. Rep..

[49] Jiang J., You Y. F., Vasu D., Chen S. C., Chiu T. W., Prashanth G., Chen P. C. (2023). Improving the p-Type CuCrO_2_ Thin Film’s
Electrical and Optical Properties. Materials..

[50] Pietro G., Benedetta A., Marcella B., Maria C. M. (2018). Raman spectroscopy
in zinc ferrites nanoparticles. Raman Spectrosc..

[51] Chiu T. W., Yu B. S., Wang Y. R., Chen K. T., Lin Y. T. (2011). Synthesis
of nanosized CuCrO_2_ porous powders via a self-combustion
glycine nitrate process. J. Alloys Compd..

[52] Lalanne M., Barnabe A., Mathieu F., Tailhades P. (2009). Synthesis
and thermostructural studies of a CuFe_1‑x_Cr_x_O_2_ delafossite solid solution with 0 ≤ ×
≤ 1. Inorg. Chem..

[53] Cai Z., Zhang F., Yu S., He Z., Cao X., Zhang L., Huang K. (2022). PBA-derived high-efficiency
iron-based
catalysts for CO_2_ hydrogenation. Catal. Sci. Technol..

[54] Torres-Ochoa J. A., Cabrera-German D., Cortazar-Martinez O., Bravo-Sanchez M., Gomez-Sosa G., Herrera-Gomez A. (2023). Peak-fitting of Cu 2p photoemission
spectra in Cu^0^, Cu^1+^, and Cu^2+^ oxides:
A method for discriminating Cu^0^ from Cu^1+^. Appl. Surf. Sci..

[55] Chen H. Y., Chang K. P., Yang C. C. (2013). Characterization
of transparent conductive
delafossite-CuCr_1–x_O_2_ films. Appl. Surf. Sci..

[56] Kameoka S., Tanabe T., Tsai A. P. (2010). Self-assembled porous
nanocomposite
with high catalytic performance by reduction of tetragonal spinel
CuFe_2_O_4_. Appl. Catal.,
A.

[57] Yu C. L., Sakthinathan S., Chen S. Y., Yu B. S., Chiu T. W., Dong C. (2021). Hydrogen generation
by methanol steam reforming process by delafossite-type
CuYO_2_ nanopowder catalyst. Microporous
Mesoporous Mater..

[58] Huang X. (2004). The influence
of Cr, Zn and Co additives on the performance of skeletal copper catalysts
for methanol synthesis and related reactions. Appl. Catal., A.

[59] Kameoka S., Okada M., Tsai A. P. (2008). Preparation of a
novel copper catalyst
in terms of the immiscible interaction between copper and chromium. Catal. Lett..

